# ESRP1-mediated biogenesis of circPTPN12 inhibits hepatocellular carcinoma progression by PDLIM2/ NF-κB pathway

**DOI:** 10.1186/s12943-024-02056-1

**Published:** 2024-07-11

**Authors:** Yang Ji, Chuangye Ni, Yanjun Shen, Zhenggang Xu, Lei Tang, Fei Yu, Lingbang Zhu, Hao Lu, Chuanyong Zhang, Shikun Yang, Xuehao Wang

**Affiliations:** 1 Hepatobiliary Center, The First Affiliated Hospital of Nanjing Medical University, Key Laboratory of Liver Transplantation, Chinese Academy of Medical Sciences, NHC Key Laboratory of Living Donor Liver Transplantation (Nanjing Medical University), No. 300 Guangzhou Road, Nanjing, Jiangsu Province 210029 China; 2 Medical College, Yangzhou University, Yangzhou, China; 3 Jiangdu People’s Hospital Affiliated to Yangzhou University, Yangzhou, China; 4 Department of General Surgery, Jinhu People’s Hospital, Huaian City, China

**Keywords:** CircPTPN12, HCC, PDLIM2, ESRP1, NF-κB pathway, Proliferation

## Abstract

**Background:**

Emerging evidence indicates the pivotal involvement of circular RNAs (circRNAs) in cancer initiation and progression. Understanding the functions and underlying mechanisms of circRNAs in tumor development holds promise for uncovering novel diagnostic indicators and therapeutic targets. In this study, our focus was to elucidate the function and regulatory mechanism of hsa-circ-0003764 in hepatocellular carcinoma (HCC).

**Methods:**

A newly discovered hsa-circ-0003764 (circPTPN12) was identified from the circbase database. QRT-PCR analysis was utilized to assess the expression levels of hsa-circ-0003764 in both HCC tissues and cells. We conducted in vitro and in vivo experiments to examine the impact of circPTPN12 on the proliferation and apoptosis of HCC cells. Additionally, RNA-sequencing, RNA immunoprecipitation, biotin-coupled probe pull-down assays, and FISH were employed to confirm and establish the relationship between hsa-circ-0003764, PDLIM2, OTUD6B, P65, and ESRP1.

**Results:**

In HCC, the downregulation of circPTPN12 was associated with an unfavorable prognosis. CircPTPN12 exhibited suppressive effects on the proliferation of HCC cells both in vitro and in vivo. Mechanistically, RNA sequencing assays unveiled the NF-κB signaling pathway as a targeted pathway of circPTPN12. Functionally, circPTPN12 was found to interact with the PDZ domain of PDLIM2, facilitating the ubiquitination of P65. Furthermore, circPTPN12 bolstered the assembly of the PDLIM2/OTUD6B complex by promoting the deubiquitination of PDLIM2. ESRP1 was identified to bind to pre-PTPN12, thereby fostering the generation of circPTPN12.

**Conclusions:**

Collectively, our findings indicate the involvement of circPTPN12 in modulating PDLIM2 function, influencing HCC progression. The identified ESRP1/circPTPN12/PDLIM2/NF-κB axis shows promise as a novel therapeutic target in the context of HCC.

**Graphical Abstract:**

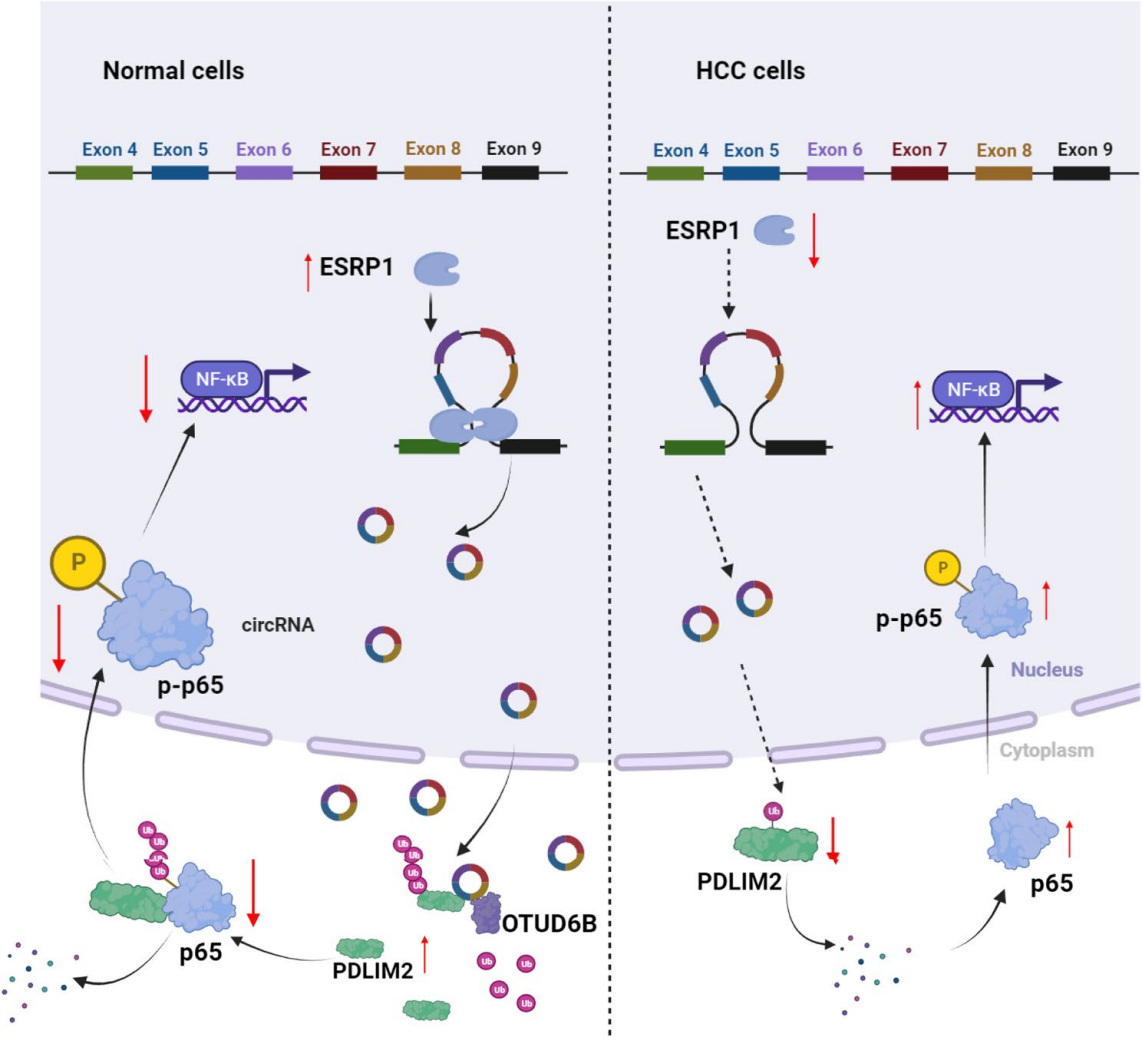

**Supplementary Information:**

The online version contains supplementary material available at 10.1186/s12943-024-02056-1.

## Introduction

Hepatocellular carcinoma (HCC), constituting 75-85% of primary liver cancer cases, ranked sixth in global cancer diagnoses and third in cancer-related mortality in 2020 [1]. Clinical staging guides therapeutic decisions for HCC patients, with early-stage cases often benefiting from curative approaches such as partial hepatectomy and liver transplantation [2]. With symptoms presenting late, advanced stages account for over 50% of HCC diagnoses, leading to the requirement for systemic therapies in these patients [3]. In the past decade, an increasing number of targeted drugs, such as Lenvatinib [4] and cabozantinib [5], have been developed. However, not all patients derive benefit from these treatments. Investigating critical targets within the genome regulatory network that influence cell survival is essential for translating molecular characterization into practical clinical applications.

Recent evidence strongly backs circular RNAs (circRNAs) as a tumorigenesis hallmark, acting as master gene modulators affecting the initiation and progression of various tumors across transcriptional and post-transcriptional epigenetic levels [6]. Compared to linear RNAs, circular RNAs lack 5′ and 3′ ends, rendering them more structurally stable. This structural characteristic expands the potential therapeutic applications of circRNAs [7]. Multiple studies indicate that circRNAs predominantly reside in the cytoplasm and perform three crucial functions: sponging miRNAs, interacting with proteins, and potentially encoding functional peptides. For example, circPLCE1 encodes a novel protein that inhibits the NF-κB signaling pathway [8]. Li et al. revealed circMTCL1’s role in stabilizing the β-catenin pathway via C1QBP interaction [9]. Additionally, Chen et al. showed circCUL2’s function as a miR-203a-3p sponge, upregulating the MyD88-dependent NF-κB pathway, fostering pancreatic ductal adenocarcinoma progression [10].

In this investigation, we characterized hsa-circ-0003764, originating from the back-splicing of protein-tyrosine phosphatase, nonreceptor-type 12 (PTPN12) [11], also named as circPTPN12, in HCC tissues. It’s specifically downregulated in HCC and acts as a novel prognostic factor in hepatocellular carcinoma. Mechanistically, circPTPN12 suppressed HCC cells proliferation while promoting apoptosis. Additionally, its interaction with PDZ-LIM domain-containing protein 2 (PDLIM2) verified circPTPN12’s capability to induce P65 degradation, thereby inhibiting HCC progression. We found circPTPN12 acts as a scaffold, binding ovarian tumor domain-containing 6B (OTUD6B) and PDLIM2, preventing PDLIM2 degradation and boosting its expression. Investigating circPTPN12’s origin, we probed the flanking intron of PTPN12 pre-mRNA. Our discovery revealed epithelial splicing regulatory protein 1 (ESRP1) binds to these intron sequences, hastening the alternative splicing of circPTPN12. This study uncovers how circPTPN12 inhibits the NF-κB pathway via its interaction with PDLIM2. Our findings introduce a new role for circPTPN12, suggesting its potential as both an effective biomarker for prognosis and a therapeutic target.

## Results

### CircPTPN12 is present within HCC tissues and exhibits downregulation

**Fig. 1:**
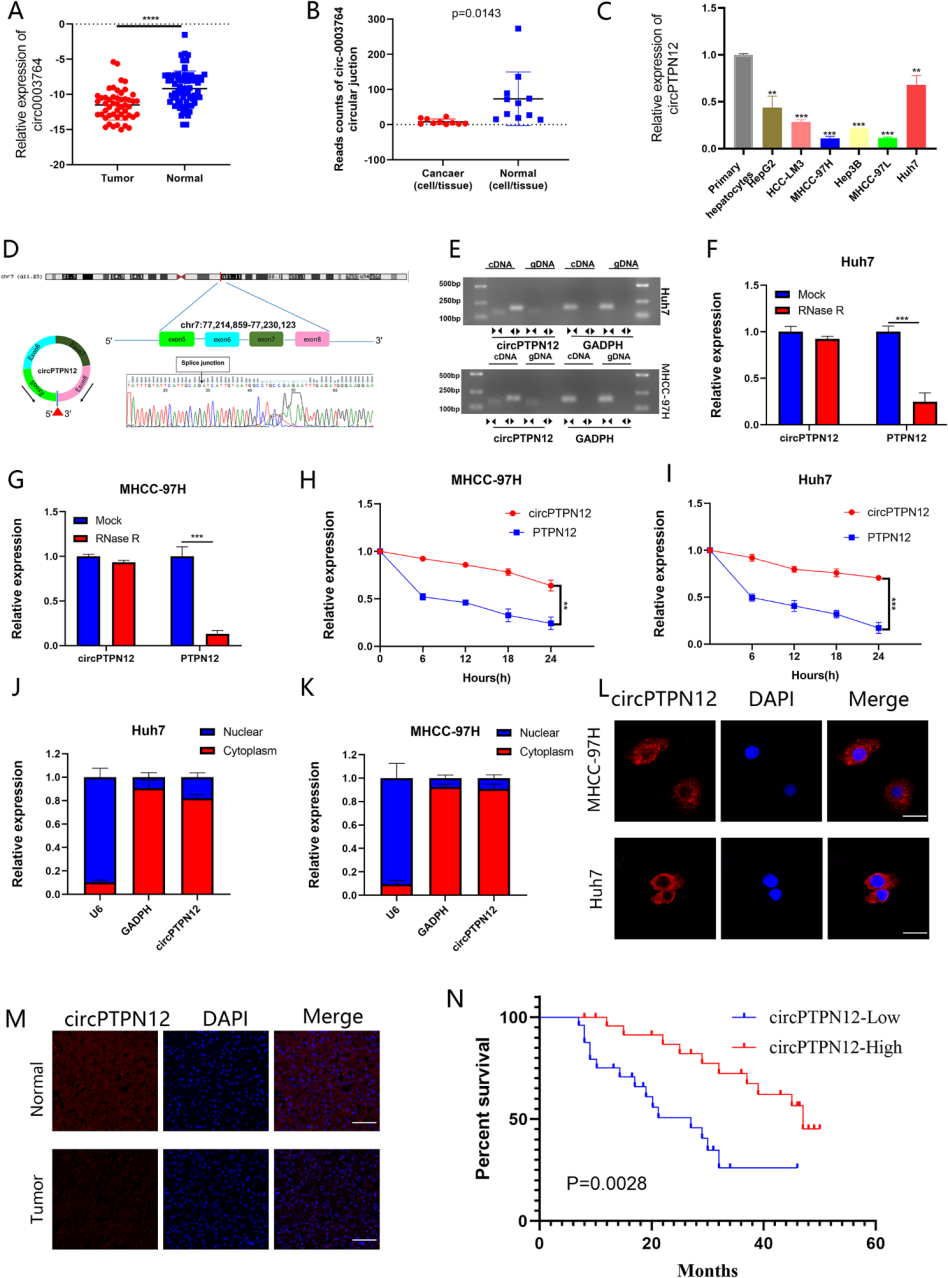
CircPTPN12 is present within HCC tissues and exhibits downregulation. (**A**) The expression of hsa-circ-0003764 in the 50 paired HCC and adjacent tissues by qRT-PCR. (**B**) Circular junction reads of hsa-circ-0003764 in normal and cancerous cells/tissues from Jeck 2013, Salzman 2013, Maass 2017 and Rybak 2015 studies in circBase. Normal cells/tissues include parietal lobe, temporal lobe, diencephalon, occipital lobe, frontal cortex, cerebellum, Hs68 RNase, A549, Ag04450, Bj, Gm12878. Cancerous cells/tissues include Helas3, Hepg2, Huvec, K562, Mcf7, Nhek, Sknshra. (**C**) The expression level of hsa-circ-0003764 in a series of cultured HCC cell lines (HepG2, HCC-LM3, MHCC-97 H, Huh7, Hep 3B and MHCC-97 L) and primary hepatocytes was analyzed by real-time PCR. (**D**) The schematic illustration showed the back splicing of circPTPN12, and sanger sequence validated the splicing site. (**E**) RT-PCR and agarose gel electrophoresis confirmed the circular formation of circPTPN12, using divergent and convergent primers in gDNA and cDNA of MHCC-97 H and Huh7 cells. GAPDH was used as a negative control. (**F, G**) CircPTPN12 and linear PTPN12 expression levels were detected after RNase R treatment in MHCC-97 H and Huh7 cells. **H, I.** CircPTPN12 and linear PTPN12 expression levels were detected after actinomycin D treatment in MHCC-97 H and Huh7 cells. **L, M.** FISH showed that the sub-cellular distribution of circPTPN12 was mostly present in the cytoplasm. L, Scale bar, 20 μm. M, Scale bar, 50 μm. **N.** Kaplan-Meier plots of the overall survival of HCC patients with high (*n* = 25) and low (*n* = 25) levels of circPTPN12. *p < 0.05; **p < 0.01; ***p < 0.001. Data were shown as mean ± SEM

PTPN12 plays a crucial role in the progression of liver cancer and other cancers [12–15]. Therefore, our attention is directed towards the 50 circRNAs derived from PTPN12 by querying the CircBase database [16]. Among them, six circRNAs have been confirmed by four independent sequencing datasets: Jeck 2013 [17], Maass 2017 [18], Rybak 2015 [19], and Salzman 2013 [20]. Utilizing qRT-PCR, we conducted an analysis to detect the expression of these six circRNAs (hsa-circ-0008139; hsa-circ-0003764; hsa-circ-0008901; hsa-circ-0002458; hsa-circ-0005197; hsa-circ-0007411) within tissues obtained from 50 patients who underwent liver cancer surgery, encompassing both cancerous and adjacent non-cancerous tissue pairs (Fig. 1A, S1A-E). The results revealed the most prominent alteration in the expression of hsa-circ-0003764. Furthermore, an analysis was conducted on the reads associated with the circ-0003764 in these four datasets. The outcomes indicated a notably lower expression of circ-0003764 in cancerous tissues/cells compared to normal tissues/cells (Fig. 1B). Additionally, validation within the GSE216613 dataset also substantiated the downregulation of circPTPN12 in HCC (Fig. S1F). Moreover, PCR results reveal diminished expression of circ-0003764 in HCC cells (HepG2, HCC-LM3, MHCC-97 H, Huh7, Hep 3B and MHCC-97 L) compared to primary hepatocytes (Fig. 1C). These data imply a potential inhibitory role of circ-0003764, urging us to further investigate its function in the context of malignant tumors in the HCC.

Hence, circ-0003764 (referred to as “circPTPN12” within this investigation) originates from exon 5–8 of the PTPN12 gene situated on chromosome 7, and its length measures 314 nucleotides as per the circBase annotation. To authenticate circPTPN12 as a genuine circRNA, we amplified its back-splice junction site using divergent primers and confirmed it through Sanger sequencing (Fig. 1D). Next, we designed divergent and convergent primers for amplifying PTPN12 and circPTPN12. Subsequent qRT-PCR analysis revealed exclusive amplification of circPTPN12 from cDNA templates using divergent primers, in contrast to the inability to achieve this from gDNA (Fig. 1E). Furthermore, qRT-PCR validation demonstrated heightened resistance of circ-PTPN12 to RNase R and actinomycin D treatments in Huh7 and MHCC- 97 H cells, as opposed to PTPN12 mRNA (Fig. 1F-I). These findings indicate that circPTPN12 exhibits a circular structure rather than a linear configuration. To further ascertain the subcellular localization of circPTPN12, both PCR and FISH techniques were employed, revealing the predominant cytoplasmic localization of circPTPN12 (Fig. 1J-L). This pattern was not only observed within cells but also extended to tissues (Fig. 1M).

In summary, circPTPN12 exhibited downregulation in HCC tissues and upregulation in corresponding adjacent non-cancerous tissues (Fig. 1A and M). Given this, an analysis linked circPTPN12 expression levels with clinicopathological data from 50 HCC patients, revealing a correlation between increased PTPN12 expression and smaller tumors size (*p* = 0.0235) and lower TNM stage(*p* = 0.0449) and Edmondson grade (*p* = 0.0277) (Table 1). HCC patients with higher circPTPN12 levels in their tumors had significantly prolonged overall survival (Fig. 1O). circPTPN12, downregulated in HCC tissues, holds promise as a marker for therapeutic strategies and prognostic evaluation in HCC.

**Table 1:** Correlation between circPTPN12 expression and clinicopathological features in HCC tissues (*n* = 50, 2 -test)

χ				
	CircPTPN12 expression			
Variables		high	low	*P*-value
		25	25	
Age (year)				0.0874
< 60		17	11	
≥ 60		8	14	
Gender				0.3946
Male		12	15	
Female		13	10	
Liver cirrhosis				0.5688
Yes		15	13	
No		10	12	
AFP (ng/mL)				0.3961
≤ 200		11	14	
> 200		14	11	
HBsAg				0.6836
positive		22	21	
negative		3	4	
Tumor size				0.0235*
< 5 cm		16	8	
≥ 5 cm		9	17	
TNM stage				0.0449*
I–II		18	11	
III–IV		7	14	
Edmondson grade				0.0277*
I–II		18	10	
III–IV		7	15	

*P < 0.05

### Effects of circPTPN12 on HCC Cell proliferation and apoptosis

To understand circPTPN12’s role in hepatocellular carcinoma (HCC), lentiviral vectors were used to overexpress or silence circPTPN12 with siRNA in MHCC-97 H or Huh7 cells. Figure S1G-H and Figure S2A demonstrate the effective modulation of circPTPN12 expression by lentiviral vectors and siRNA, while the specificity of PTPN12 mRNA expression remained unaltered. Cell Counting Kit-8 (CCK-8) assays were used to assess the biological effects of circPTPN12 knockdown and overexpression. Overexpressing circPTPN12 in MHCC-97 H cells notably reduced their growth rate, while silencing circPTPN12 in Huh7 cells enhanced their growth rate (Fig. S2B). Additionally, colony formation assays (Fig. S2C-D) and EdU assays indicated decreased proliferation in MHCC-97 H cells with circPTPN12 overexpression and increased proliferation in Huh7 cells with circPTPN12 knockdown (Fig. S2E-F). Apoptosis assays revealed lower apoptotic rates in HCC cells with circPTPN12 siRNA and increased rates in MHCC-97 H cells overexpressing circPTPN12 (Fig.S2G-H). The levels of apoptosis- related proteins varied in accordance with the changes in circPTPN12 expression (Fig. S2I). BCL2 decreases when circPTPN12 is overexpressed, while bax increases under the same conditions. The knockdown group exhibits the opposite effects. Overall, circPTPN12 inhibits HCC cell proliferation and promotes apoptosis in vitro.

### CircPTPN12 inhibits the progression of HCC through NF-κB pathway by promoting P65 degradation

**Fig. 2:**
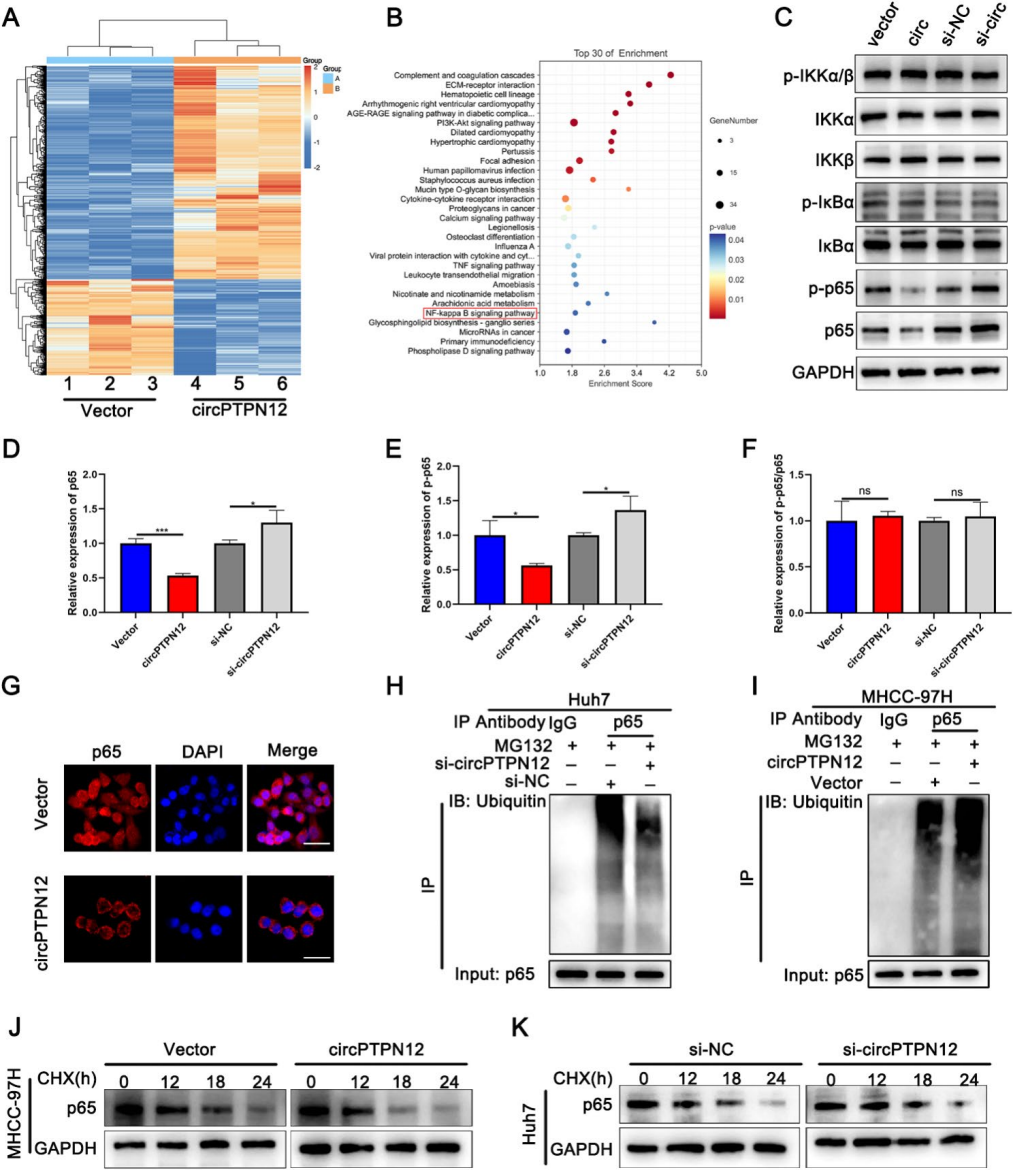
CircPTPN12 inhibits the progression of HCC through NF-κB pathway by promoting p65 degradation. (**A**) Heatmap showed the RNA-seq result in circPTPN12 overexpression and control MHCC-97 H cells. (**B**) Pathway enrichment analysis of differentially expressed genes in RNA-sequence data. (**C**) Western blot analyses of the levels of p-IKKα/β, p-IκBα, p-p65, total IKKα/β, IκBα and p65 after upregulating or downregulating circPTPN12 in HCC cells. (**D**) The protein level of p65 in MHCC-97 H and Huh7 cells after upregulating or downregulating circPTPN12. (**E**) The protein level of p-p65 in MHCC-97 H and Huh7 cells after upregulating or downregulating circPTPN12. (**F**) The protein level of p-p65/p65 in MHCC-97 H and Huh7 cells after upregulating or downregulating circPTPN12. (**G**) Immunofluorescence assay evaluating the p65 in MHCC-97 H cells. Scale bar, 20 μm. **H, I.** The ubiquitination level of p65 in MHCC-97 H and Huh7 cells after upregulating or downregulating circPTPN12. **J, K.** Western blot analysis of P65 in MHCC-97 H and Huh7 cells treated with CHX treatment. *p < 0.05; **p < 0.01; ***p < 0.001. Data were shown as mean ± SEM

To further elucidate the regulatory mechanism of circPTPN12 in hepatocellular carcinoma (HCC) progression, we conducted RNA sequencing on MHCC-97 H cells transfected with lentivirus overexpressing circPTPN12. The results revealed a total of 1372 downregulated and 618 upregulated genes. Enrichment analysis of the downregulated genes unveiled suppression of the NF-κB signaling pathway upon overexpression of circPTPN12 (Fig. 2A-B). In the context of this study, Western blot analysis revealed that elevated expression of circPTPN12 led to a decrease in P65 protein levels, while not influencing the phosphorylated P65 (p-p65) levels (Fig. 2C-F). Immunofluorescence further validated these findings (Fig. 2G). These findings strongly indicate that circPTPN12 exerts its regulatory effect on the NF-κB pathway through modulation of P65 protein expression. Further investigations revealed that circPTPN12 does not exert an influence on the transcription of the P65 gene (Fig. S3A). Therefore, we postulate that circPTPN12 modulates the protein levels of P65 through its impact on post-transcriptional modifications of P65. Subsequent results demonstrated that circPTPN12 promotes the ubiquitination of P65 (Fig. 2H-I). We specifically evaluated the protein expression of P65 in the presence of cycloheximide (CHX), a protein synthesis inhibitor. Over time, a decline in the protein levels of P65 was observed (Fig. 2J-K). To delve deeper into the dependence of circPTPN12’s tumor-inhibitory impact on P65, we treated MHCC-97 H cells overexpressing circPTPN12 with P65 siRNA. Interestingly, the prominent tumor-inhibitory effect of circPTPN12 on HCC was notably attenuated (Fig. S3B-J). Our findings collectively indicate that circPTPN12 curtails the progression of HCC by targeting P65, resulting in the deactivation of the NF-κB signaling pathway.

### CircPTPN12 associates with PDLIM2 in HCC cells

**Fig. 3:**
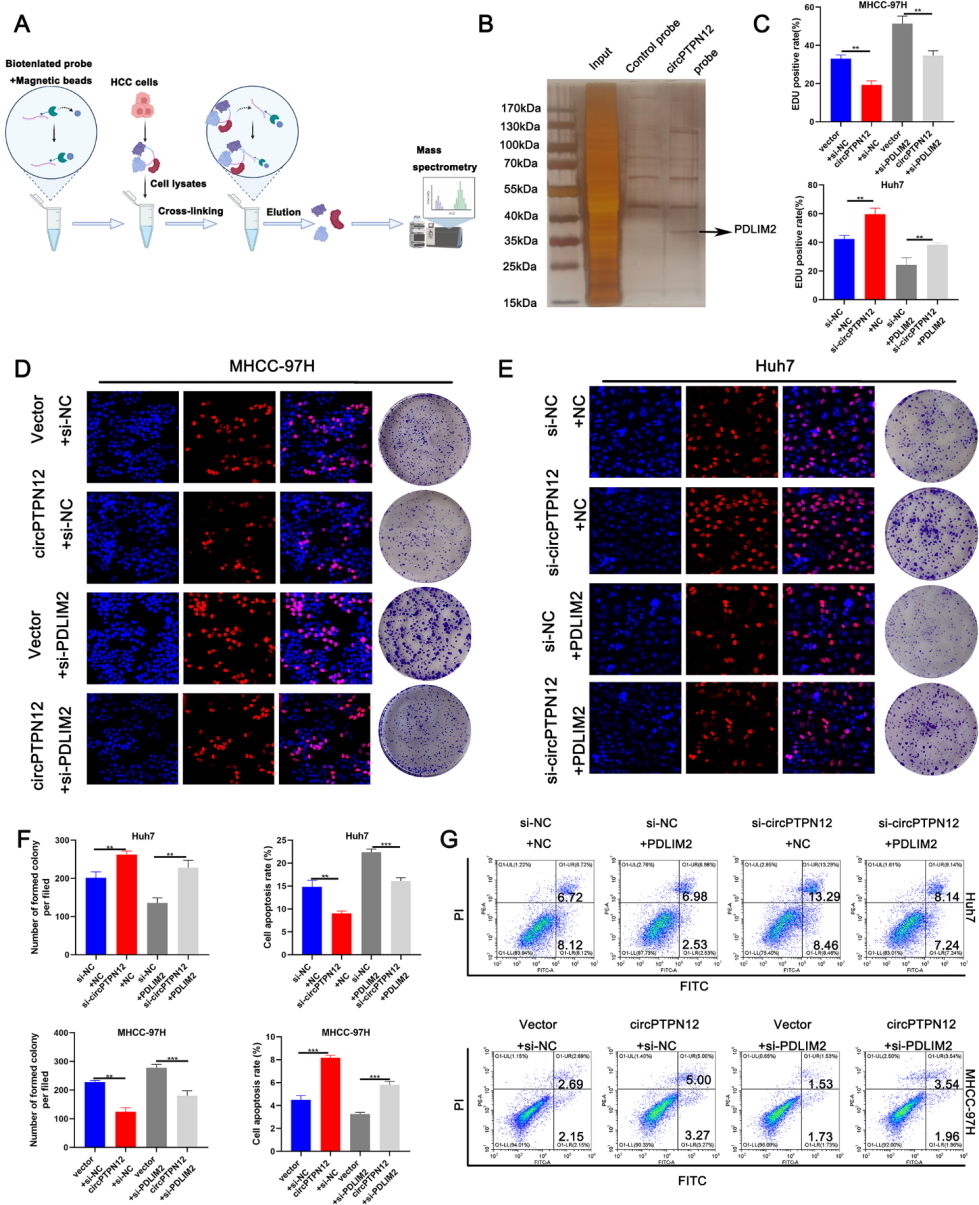
CircPTPN12 associates with PDLIM2 in HCC cells. (**A**) Schematic diagram of RNA pulldown using the circPTPN12 probe and subsequent mass spectrometry assay. (**B**) Silver-stained SDS-PAGE gel-containing proteins derived from RNA pulldown by circPTPN12 probe and negative control. **C-F**. EdU and colony formation assays were carried out for rescue experiments. 3D, the restoring effect of si-PDLIM2 on circPTPN12 upregulation in MHCC-97 H cells; 3E, the restoring effect of ectopic PDLIM2 expression on circPTPN12 downregulation in Huh7 cells. Scale bar, 50 μm. **G.** Cell apoptosis was determined after cell transfection. *p < 0.05; **p < 0.01; ***p < 0.001. Data were shown as mean ± SEM

Based on existing theoretical research [21], cytoplasmically localized circRNAs have been found to participate in post-transcriptional regulation through mechanisms such as competing endogenous RNAs (ceRNAs), protein coding, and protein binding, thereby exerting significant biological effects. To elucidate the precise mechanisms underlying the physiological functions of circPTPN12, we performed RNA pulldown assays employing a biotin-labeled probe (Fig. 3A-B). Argonaute 2 protein (Ago2), ubiquitously expressed in organisms, serves as a pivotal constituent of the RNA-induced silencing complex, contributing to miRNA-mediated sponge functions [22]. Given the absence of Ago2 identification in the mass spectrometry data (Table S4), we ruled out the miRNA sponge function of circPTPN12. The online databases circBank and circRNADb suggested the potential of circPTPN12 in encoding functional peptides (Fig. S4A). Supplementary Fig. 4B details critical attributes such as the internal ribozyme entry site (IRES), open reading frame (ORF), as well as the translation start and termination sites of PTPN12-104aa. Plasmids carrying the wild-type (WT) sequence, featuring ATG as the translation initiation site, were engineered to verify the presence of the PTPN12-104aa protein within HCC cells. The data depicted in Supplementary Fig. 4C definitively exclude the possibility of circPTPN12 encoding a functional peptide in vitro. Drawing upon these experimental findings, we ascertain that circPTPN12 neither functions as a microRNA (miRNA) sponge nor encodes peptides.

Then, the outcomes of MS unveiled the specific binding of 73 proteins to the circPTPN12 probe (Table S4). Moreover, in contrast to the circPTPN12 control probe, the protein PDLIM2 emerged as a particularly interesting candidate (Fig. 3A-B). Furthermore, supported by its function as a ubiquitin ligase [23], enabling P65 degradation, our rescue experiment effectively illustrates that circPTPN12 operates through PDLIM2 to facilitate P65 degradation (Fig. S4D-G), consequently reducing P65 protein levels without affecting its transcriptional levels (Fig. S4H-I). Furthermore, we conducted an analysis of the PDLIM2 interaction network utilizing the Search Tool for the Retrieval of Interacting Genes/Proteins (STRING) database. Our findings revealed a correlation between the P65 protein and PDLIM2 within this network (Fig. S4J). Typically, circRNAs act as molecular scaffolds for proteins, facilitating their interaction and enhancing the function of ubiquitin ligases. However, intriguingly, the mass spectrometry (MS) results of circRNA did not indicate the presence of P65. This initiated a comprehensive investigation into the underlying mechanisms by which circRNA regulates P65 protein levels through PDLIM2, thereby influencing the functional aspects of the NF-κB pathway.

### PDLIM2 is the functional downstream mediator of circPTPN12

In order to investigate whether circPTPN12 exerts its functions through the regulation of PDLIM2 expression, we conducted further experiments involving the utilization of siRNAs and plasmids for PDLIM2 knockdown and overexpression (Fig. S5B). As depicted in Fig. 3C-G, our results demonstrated a significant reversal of the effects on cell proliferation and apoptosis in Huh7 cells due to the introduction of ectopic PDLIM2 expression following circPTPN12 downregulation. Likewise, the impact of circPTPN12 overexpression on cell malignancy was counteracted by PDLIM2 knockdown in MHCC-97 H cells (Fig. 3C-G, Fig. S5C-D). Additionally, we validated the protein levels of PDLIM2 in liver cancer tissues using a database (https://cprosite.ccr.cancer.gov/), further confirming its relatively lower expression in cancerous tissues compared to adjacent non-cancerous tissues (Fig. S5E). These findings strongly imply that circPTPN12 plays a role in inhibiting HCC cell malignancy through the upregulation of PDLIM2 expression.

### cicrPTPN12 inhibits the degradation of PDLIM2

**Fig. 4:**
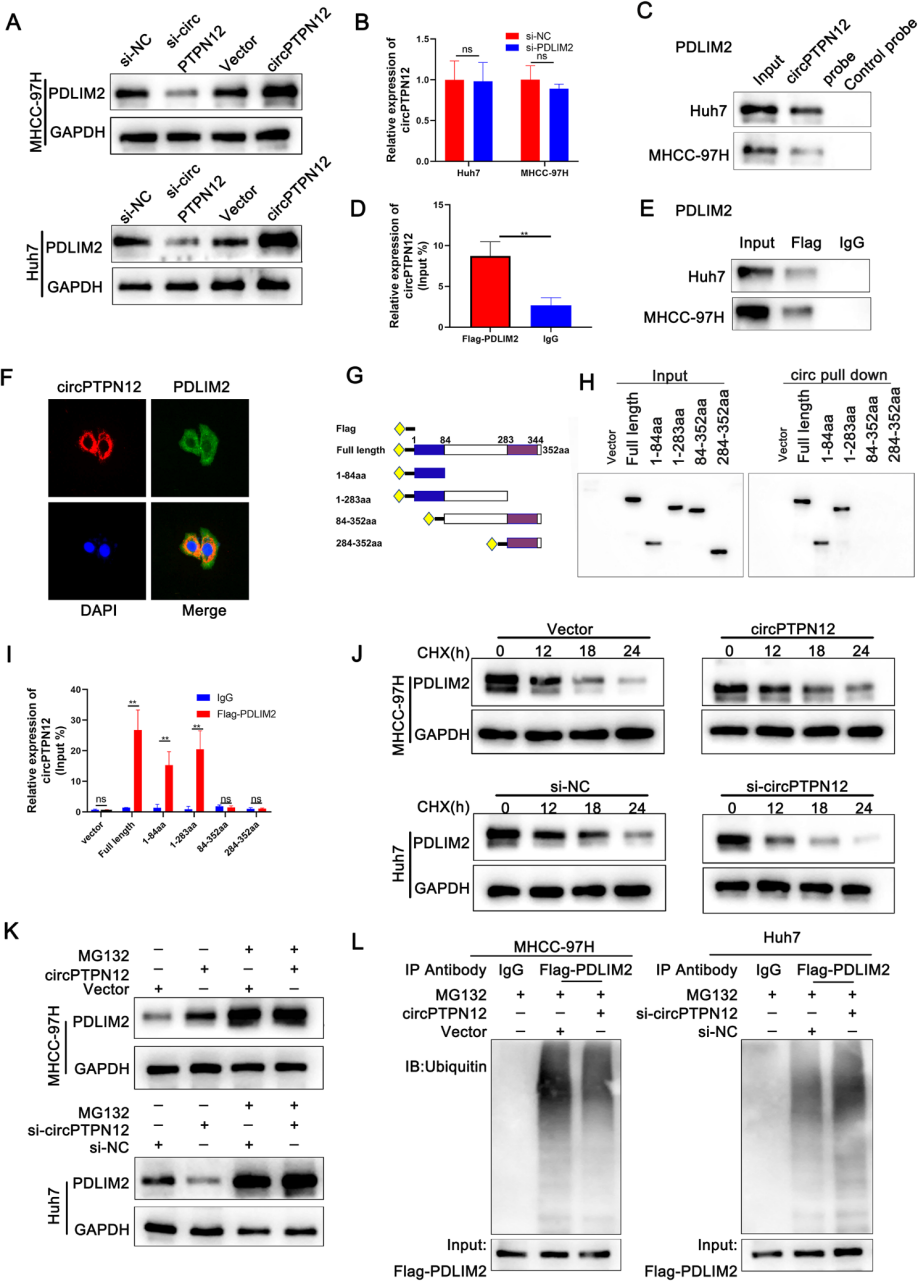
CicrPTPN12 inhibits the degradation of PDLIM2. **A**. Western blot analysis showed the association of circPTPN12 with PDLIM2. **B**. CircPTPN12 expression was determined after knocking down PDLIM2 by qRT-PCR assay. **C.** Western blot assays showing the effect of circPTPN12 on the proteins pulled down by circPTPN12 probe. **D-E.** RIP assay indicated the association between circPTPN12 and PDLIM2. **F.** RNA FISH and immunofluorescence analysis indicated the colocalization of PDLIM2 (green) and circPTPN12 (red) in Huh7 cells. Scale bar, 20 μm. **G.** Functional domain and truncated mutation annotation of PDLIM2. **H-I.** RIP assay and RNA pulldown assay confirmed that circPTPN12 characteristically interacted with the PDZ domain of PDLIM2. The blue segment represents the PDZ domain, while the purple segment represents the LIM domain. **J.** Western blot analysis of PDLIM2 in MHCC-97 H and Huh7 cells treated with CHX treatment. **K.** Protein level of PDLIM2 in HCC cells treated with 20µM MG132 for 12 h. Upper, MHCC-97 H cells transfected with lentivirus for circPTPN12 overexpression and control; down, Huh7 cells transfected with circPTPN12 siRNA and control. **L.** Ubiquitination modification of PDLIM2 proteins in HCC cells was determined via Co-IP and western blotting. *p < 0.05; **p < 0.01; ***p < 0.001. Data were shown as mean ± SEM

Given the experimental findings mentioned above, we were intrigued and decided to further explore PDLIM2 potential interaction with circPTPN12. In addition, Western blot (WB) experiments revealed that PDLIM2 exhibited alterations in response to changes in circPTPN12 levels within both Huh7 and MHCC-97 H cells (Fig. 4A). In contrast, circPTPN12 did not alter PDLIM2 transcriptional levels (Fig. S5F). Additionally, depicted in Fig. 4B and Figure S5G, altering PDLIM2 expression levels did not affect the expression of circPTPN12. This suggests a potential scenario: circPTPN12 may impact P65 degradation and regulate the NF-ΚB pathway by modulating PDLIM2 protein levels. To scrutinize this interaction further, we employed WB, uncovering the presence of PDLIM2 within the protein complexes obtained from the circPTPN12 pull-down assay (Fig. 4C). The RNA immunoprecipitation (RIP) assay, employing a PDLIM2-specific antibody, confirmed the recruitment of circPTPN12 by PDLIM2. (Fig. 4D-E). Furthermore, we conducted structural predictions to assess the potential interaction between circPTPN12 and PDLIM2. Utilizing the secondary structure of circPTPN12 characterized by the lowest free energy, we constructed three-dimensional (3D) models depicting the complex formation between circPTPN12 and PDLIM2 (PDB: 3PDV) (Fig. S5H). The co-localization of circPTPN12 with PDLIM2 was subsequently verified through immunofluorescence staining in Huh7 cells. It was observed that they predominantly co-existed within the cytoplasm (Fig. 4F). Further domain mapping assays demonstrated that the PDZ domain (1-84aa) of PDLIM2 protein played a crucial role in binding to circPTPN12 (Fig. 4G-I), thus indicating a direct interaction between circPTPN12 and PDLIM2. Given that reduced protein levels can arise from decreased synthesis or shortened half-life, we investigated How circPTPN12 could influence the stability of the PDLIM2 protein. We performed a series of experiments to assess the expression of PDLIM2 under diverse conditions. Specifically, we assessed the protein expression of PDLIM2 in the presence of cycloheximide (CHX), an inhibitor of protein synthesis. As time progresses, there is a decrease in the protein levels of PDLIM2 (Fig. 4J). The elevation of PDLIM2 protein levels induced by circPTPN12 overexpression could be reversed by MG132. The reduction in PDLIM2 protein levels resulting from circPTPN12 knockdown in Huh7 cells was restored by MG132 (Fig. 4K). Co-immunoprecipitation (Co-IP) and Western blotting analyses revealed that the depletion of circPTPN12 in Huh7 cells elicited a marked elevation in the ubiquitination status of PDLIM2. Conversely, the introduction of circPTPN12 overexpression impeded the ubiquitination process of PDLIM2 in MHCC-97 H cells. (Fig. 4L). Collectively, these findings imply that circPTPN12 potentially rescues PDLIM2 from undergoing proteasomal degradation.

### OTUD6B as a specific deubiquitinating enzyme involved in PDLIM2 deubiquitination

**Fig. 5:**
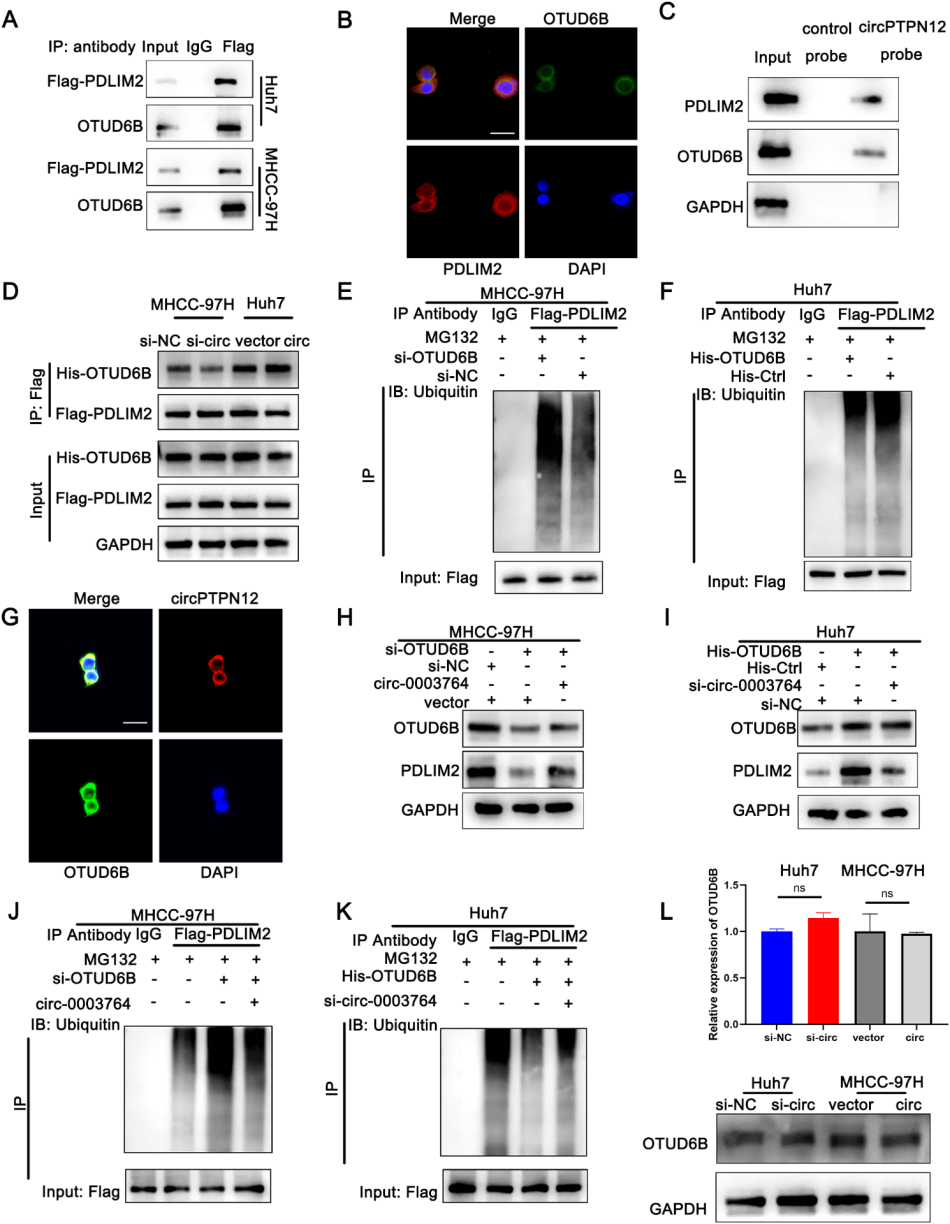
OTUD6B as a specific deubiquitinating enzyme involved in PDLIM2 deubiquitination. (**a**) Co-IP with Flag antibody confirmed the existence of OTUD6B in the precipitates of Flag-PDLIM2. (**B**) Immunofluorescence analysis of the localization of PDLIM2 (red) and OTUD6B (green). Scale bar, 20 μm. (**C**) RNA pull-down confirmed the binding of circPTPN12 and OTUD6B. (**D**) Plasmids encoding His-OTUD6B and Flag-PDLIM2 were co-transfected into MHCC-97 H and Huh7 cells for 24 h, followed by immunoprecipitation with antibody against Flag or with control IgG. Immunoprecipitates and cell lysates were western blotting detected with antibodies against the indicated proteins. (**E**) RNA FISH and immunofluorescence analysis of the localization of circPTPN12 (red) and OTUD6B (green). Scale bar, 20 μm. **F-G.** PDLIM2 ubiquitination was detected after OTUD6B knockdown or overexpression. 5 F, Huh7 cells; 5G, MHCC cells. **H.** After circPTPN12 overexpression, western blotting detected the PDLIM2 protein level in HCC cells with OTUD6B knockdown. **I.** After circPTPN12 knockdown, western blotting detected the PDLIM2 protein level in HCC cells with OTUD6B overexpression. **J.** Ubiquitination modification of PDLIM2 in MHCC-97 H cells after OTUD6B knockdown and circPTPN12 overexpression detected via Co-IP and western blotting. **K.** Ubiquitination modification of PDLIM2 in Huh7 cells after OTUD6B overexpression and circPTPN12 knockdown detected via Co-IP and western blotting. **L**. QRT-PCR and western blotting detected the OTUD6B expression in HCC cells with circPTPN12 knockdown or overexpression. *p < 0.05; **p < 0.01; ***p < 0.001. Data were shown as mean ± SEM

Subsequently, we investigated the potential mechanism by which circPTPN12 regulates the ubiquitination of PDLIM2. The mass spectrometry results were further examined, revealing that the circPTPN12 probe successfully captured OTUD6B (Fig. S5I), which was previously identified as a deubiquitinating enzyme [24]. Consistently, a Co-IP assay revealed the binding of OTUD6B to PDLIM2 (Fig. 5A). Based on these findings, we propose the hypothesis that circPTPN12 facilitates the deubiquitination of PDLIM2 by binding to OTUD6B. Additionally, immunofluorescence staining of OTUD6B and PDLIM2 confirmed their colocalization in HCC cells (Fig. 5B). RNA pull-down assays confirmed the association between PDLIM2, OTUD6B and circPTPN12 (Fig. 5C). Additionally, the Co-immunoprecipitation (Co-IP) assay showed a decrease in the binding between OTUD6B and PDLIM2 in circPTPN12 knockdown cells compared to control cells. Conversely, circPTPN12 overexpression had the opposite effect, increasing their binding (Fig. 5D). RNA FISH and immunofluorescence analysis confirmed the colocalization of OTUD6B with circPTPN12 within the cytoplasm (Fig. 5E). Then, we employed an online database (SWISS-MODEL Interactive Workspace; https://swissmodel.expasy.org/interactive) to predict the 3D structure of OTUD6B and utilized HDOCK to predict the potential binding between circRNA and OTUD6B. (Fig. S5H). These findings imply that the OTUD6B protein may form interactions with both the PDLIM2 protein and circPTPN12.

For a deeper exploration of OTUD6B’s influence on PDLIM2, Western blot findings revealed that the upregulation of OTUD6B correlated with elevated PDLIM2 protein levels, whereas the downregulation of OTUD6B corresponded to a decrease in PDLIM2 protein expression (Fig. S5J). Immunoprecipitation (IP) results indicated that the knockdown of OTUD6B effectively enhanced the ubiquitylation of PDLIM2, while overexpression of OTUD6B decreased the ubiquitylation of PDLIM2 (Fig. 5F-G). The experimental results demonstrated that overexpressing OTUD6B notably increased PDLIM2 protein levels in HCC cells. This increase could be counteracted by circPTPN12 knockdown. Conversely, reducing OTUD6B levels led to a decrease in PDLIM2 protein levels, and this effect was enhanced by ectopic circPTPN12 expression (Fig. 5H-I). Co-IP assays and western blotting indicated that the ubiquitination of PDLIM2, hindered by OTUD6B overexpression, was notably reduced by circPTPN12 knockdown. Conversely, reducing OTUD6B levels and further enhancing the ubiquitination of PDLIM2 was observed upon circPTPN12 overexpression (Fig. 5J-K). Meanwhile, circPTPN12 did not affect the levels of OTUD6B itself (Fig. 5L). These findings indicate that circPTPN12 acts as a scaffold between PDLIM2 and OTUD6B, promoting their interaction and preventing the ubiquitination of PDLIM2. This ultimately leads to the inhibition of PDLIM2 degradation.

### Tumor suppressor ESRP1 is involved in the generation of circPTPN12

**Fig. 6:**
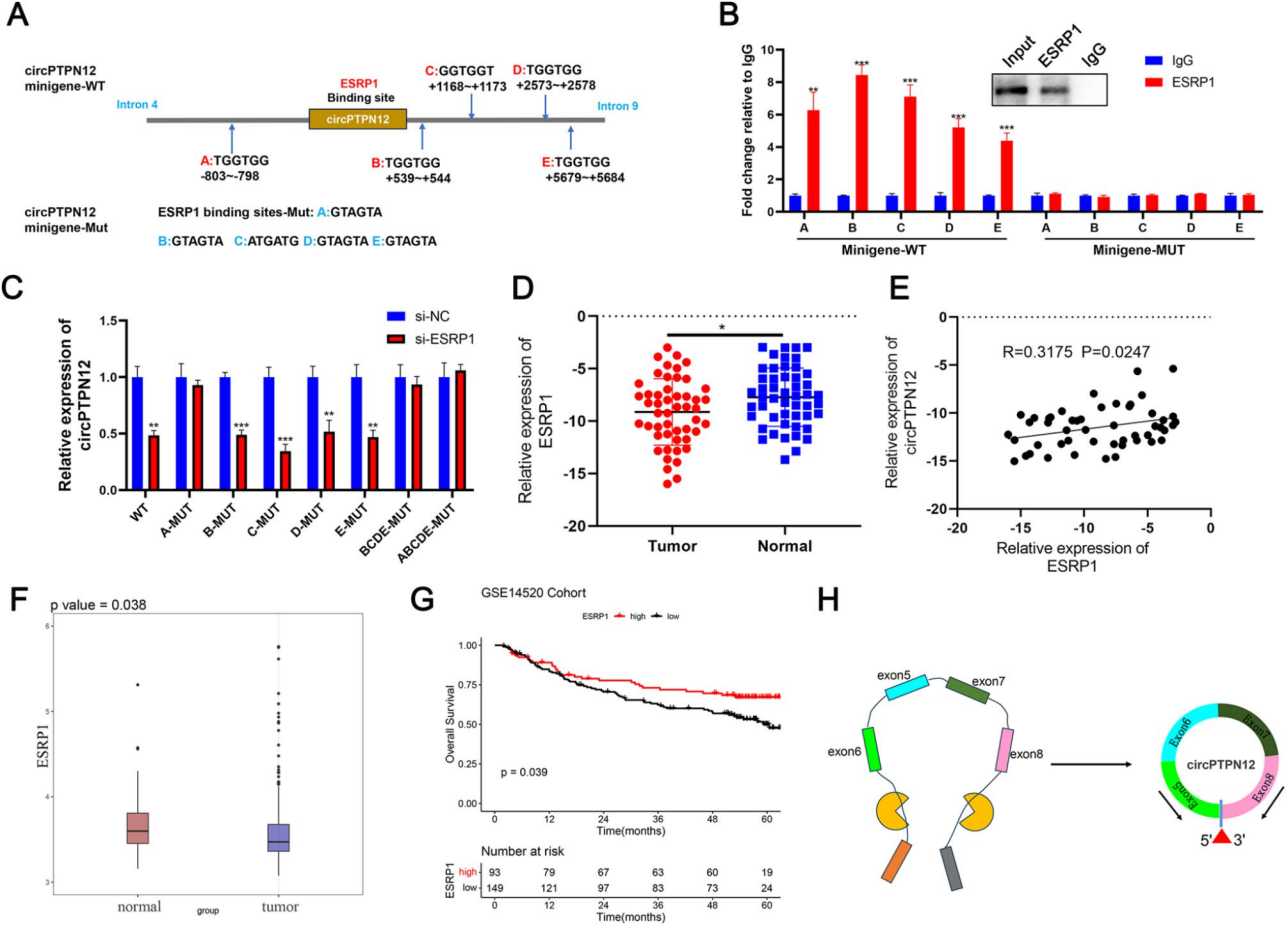
Tumor suppressor ESRP1 is involved in the generation of circPTPN12. (**a**) Multiple ESRP1 binding sites were observed in the flanking of circPTPN12. (**B**) The binding of ESRP1 to circPTPN12 flanks was verified by RNA immunoprecipitation (RIP) experiments. (**C**) QRT-PCR showed the circPTPN12 expression in Huh7 cells transfected with ESRP1 silencing shRNA or controls with these mutant sequences (one motif mutant). (**D**) QRT-PCR analysed the mRNA levels of ESRP1 in HCC tissues. (**E**) The correlation between the expression of ESRP1 and circPTPN12 in HCC tissues. (**F**) Analysis of mRNA levels of ESRP1 in HCC from GSE14520 datasets. (**G**) Kaplan-Meier curves displaying the impact of ESRP1 on overall survival in the HCC cohort from GSE14520 datasets. (**H**) Schematic diagram showed ESRP1 promotes the biogenesis of circPTPN12. *p < 0.05; **p < 0.01; ***p < 0.001. Data were shown as mean ± SEM

Prior research has demonstrated the interaction of RNA binding proteins (RBPs) like QKI [25] and FUS [26] with the upstream and downstream flanking sequences of circRNAs within pre-mRNAs, thereby facilitating the cyclization process of circRNAs. Based on the MS results binding upstream and downstream flanking sequences of circPTPN12 (Table. S5), we identified ESRP1 as a typical splicing factor involved in the formation of circRNAs (Fig. S6A). In order to assess the significance of ESRP1-binding sequences in the formation of circPTPN12, we conducted a search for GGT-rich sequences, known as ESRP1-binding motifs [27], within the flanking intronic regions of circPTPN12. This exploration led to the identification of five potential ESRP1-binding sites (Fig. 6A). Subsequently, we generated wild-type and mutant circPTPN12 minigenes for the purpose of conducting a RIP assay. Our findings revealed that ESRP1 exhibited binding affinity specifically to the putative wild-type binding sites situated within the flanking introns, while showing no affinity towards the mutant sites (Fig. 6B). Furthermore, our observations revealed that, in comparison to si-NC, the knockdown of ESRP1 significantly reduced circPTPN12 production in HCC cells transfected with wild-type circPTPN12 minigenes or those carrying mutations individually, except for the A mutation. Conversely, ESRP1 knockdown exhibited negligible or minimal impact on circPTPN12 production in cells transfected with minigenes carrying mutations in A or (and) B / C / D / E—highlighting the essential nature of these five motifs within the flanking introns for ESRP1-mediated circPTPN12 biogenesis (Fig. 6C). Furthermore, the RT-PCR analysis revealed elevated levels of linear transcript expression (exon 4–5, exon 5–8, and exon 8–9) following ESRP1 knockdown compared to the ESRP1 control transfection (Fig. S6B). Clinically, our investigation unveiled lower expression levels of ESRP1 in HCC tissues, with a positive correlation observed between ESRP1 expression and circPTPN12 levels in these tissues (Fig. 6D-E). This finding aligns with the GEO database (GSE14520) (Fig. 6F). Furthermore, HCC patients exhibiting low ESRP1 expression demonstrated significantly poorer overall survival rates (*p* = 0.039) (Fig. 6G). Collectively, these data underscore the potential role of the splicing factor ESRP1 in enhancing circPTPN12 biogenesis through its targeted interaction with the flanking intronic regions (Fig. 6H). These findings collectively indicate the existence of a ESRP1-mediated circPTPN12/PDLIM2/NF-κB axis in HCC, capable of inducing apoptosis and suppressing cellular proliferation.

### CircPTPN12 inhibits the growth of HCC cell in vivo

**Fig. 7:**
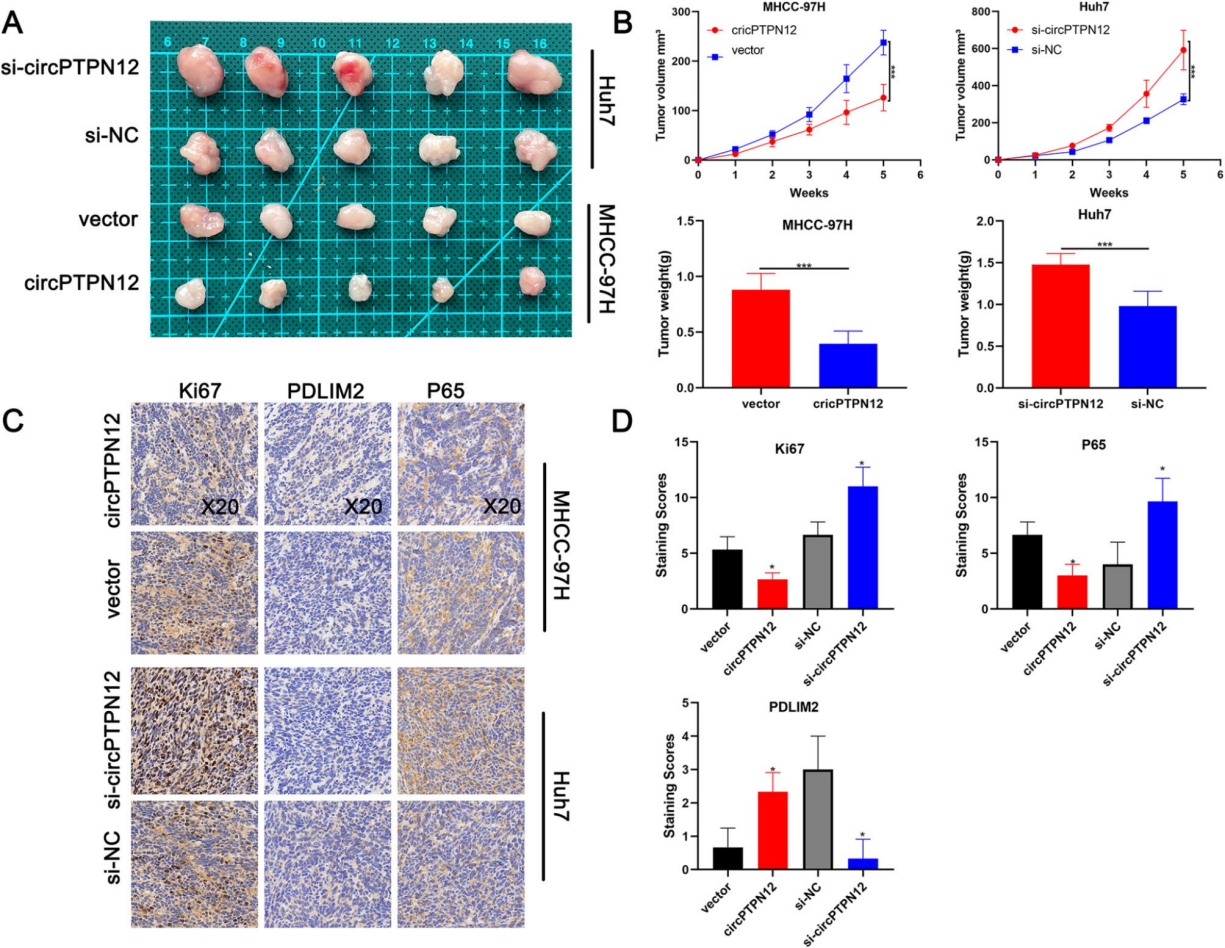
CircPTPN12 inhibits the growth of HCC cell in vivo. **A, B.** Tumor size and weight of the subcutaneous tumor growth model injected with HCC cells (5 mice/group). **C**. IHC staining of the indicated proteins in the tumor samples obtained from the subcutaneous tumor growth model. Left, Ki-67; middle, PDLIM2; right, p65. **D.** IHC staining scores of xenograft tumors. *p < 0.05; **p < 0.01; ***p < 0.001. Data were shown as mean ± SEM

To assess the impact of circPTPN12 on HCC cell growth in vivo, we conducted subcutaneous injections of HCC cells into nude mice. Results depicted in Fig. 7A and B revealed a notable deceleration in tumor growth rate and reduced tumor weight in xenograft mouse models with circPTPN12 overexpression, while circPTPN12 knockdown in HCC cells yielded contrasting effects. Immunohistochemistry (IHC) analysis was subsequently performed to validate the in vivo effect of circPTPN12. The distribution of Ki67 was significantly lower in tumors exhibiting circPTPN12 overexpression compared to the control, whereas circPTPN12 knockdown elicited the opposite effects. Moreover, IHC analysis affirmed a substantial reduction in PDLIM2 protein expression following circPTPN12 knockdown, while an opposing trend was observed upon circPTPN12 overexpression. Additionally, the expression levels of P65 exhibited an inverse correlation with PDLIM2 expression (Fig. 7C-D). Taken together, these findings collectively indicate the potent inhibitory effect of circPTPN12 on tumor growth in vivo.

## Discussion

Circular RNAs (circRNAs) have emerged as crucial contributors to disease pathogenesis, notably in cancer [28]. Our study identified circPTPN12 as a novel circRNA exhibiting tumor-suppressive properties in hepatocellular carcinoma (HCC). Remarkably, reduced circPTPN12 expression was detected in tumor tissues of individuals with HCC, exhibiting a correlation with larger tumor size, advanced TNM stage, higher Edmondson grade, and an unfavorable prognosis. Meanwhile, in both in vivo and in vitro experiments, the overexpression of circPTPN12 significantly inhibited the growth of HCC. This highlights the potential of circPTPN12 as a prognostic indicator for HCC. Then, we elucidated the tumor-suppressive mechanism of circPTPN12, primarily involving the inhibition of NF-κB signaling. Firstly, circPTPN12 facilitated P65 degradation through PDLIM2. Secondly, by interacting with the OTUD6B protein, circPTPN12 inhibited PDLIM2 ubiquitination. Interestingly, elevation in ESRP1 expression facilitated its interaction with the flanking introns of circPTPN12, resulting in increased circPTPN12 production. Thus, our findings identify that circPTPN12 inhibits HCC proliferation and promoting apoptosis, which advance the understanding of molecular mechanism involved in HCC.

The pathogenesis of hepatocellular carcinoma (HCC) is characterized by continual hepatocyte demise, infiltration of inflammatory cells, and the compensatory regeneration of liver tissue. Meanwhile, NF-κB pathway which has been identified as a pivotal role in those progression [29]. In recent years, circRNA has emerged as a novel focus in RNA therapeutics [7]. For instance, circADAMTS6 positively regulates NF-κB signaling in glioblastoma by inhibiting ANXA2 degradation, particularly in hypoxic microenvironments [30]. Shan et al. demonstrated that circCYP24A1 interaction with PKM2 activates NF-κB signaling, contributing to esophageal squamous cell carcinoma progression [31]. Additionally, Cao et al. reported that circCORO1C activates the NF-κB pathway, enhancing the expression of PDL1 and promoting liver cancer proliferation and metastasis in HCC [32]. In this study, we conducted transcriptomic RNA sequencing to investigate the mechanism underlying circPTPN12’s inhibition of malignant progression in liver cancer. When circPTPN12 is overexpressed, enrichment analysis indicates the suppression of the NF-κB signaling pathway. Ubiquitination, a reversible enzymatic cascade, serves as a crucial posttranslational modification process. Our findings demonstrate that circPTPN12 modulates the posttranslational modification of P65, reducing its protein stability. This, in turn, inhibits the malignant progression of hepatocellular carcinoma (HCC), promoting apoptosis and suppressing cell proliferation. However, the phosphorylation modification process of P65 remains largely unaltered in this context. Thus, our discovery unveils a novel mechanism wherein circPTPN12 exerts its cancer-inhibiting effect by destabilizing oncogenic proteins.

Typically, circRNAs exert their functional mechanisms by serving as miRNA sponges [33], interacting with proteins [34], and encoding functional peptides [35]. In our study, to investigate the mechanism by which circPTPN12 facilitates P65 ubiquitination, excluding the other two functional mechanisms, we identified, through mass spectrometry experiments. PDLIM2, alternatively known as Mystique or SLIM, is categorized within the actinin-associated LIM protein family due to its possession of PDZ and LIM domains [36]. PDLIM2 has been established as a tumor suppressor and serves as a ubiquitin ligase for P65 [23]. Especially in hepatocellular carcinoma (HCC), Jiang and colleagues illustrated a reduction in PDLIM2 expression in both tissue samples and cells, which was associated with an unfavorable prognosis for individuals diagnosed with HCC [37]. The experimental findings revealed that circPTPN12 facilitates P65 ubiquitination via PDLIM2, consequently inhibiting P65 protein levels. Typically, circRNAs serve as scaffolds for functionality. Additionally, PDLIM2, known as a tumor suppressor, exhibits low expression in liver cancer tissues. However, contrary to expectations, mass spectrometry results did not detect binding between circPTPN12 and P65. Furthermore, circPTPN12 did not alter P65 transcription levels. Hence, these observations prompted further investigation into the specific mechanism by which circPTPN12 functions through PDLIM2.

An increasing body of research highlights that the ubiquitination status of proteins results from the coordinated actions of ubiquitin ligases and deubiquitinating enzymes (DUBs). Deubiquitinating enzymes play crucial roles in regulating fundamental biological processes, including DNA repair, apoptosis, oncogene function, and checkpoint control. OTUD6B identified as a deubiquitinating enzyme, has been shown to deubiquitinate PVHL, inhibiting tumor progression in clear cell renal cell carcinoma [24]. In our investigation, immunofluorescence assays revealed the cellular localization of both circPTPN12 and PDLIM2 within the cytoplasm. We observed that circPTPN12 interacts with the PDZ domain of PDLIM2. Consequently, alterations in PDLIM2 protein levels were noticed upon the expression of circPTPN12. Moreover, circPTPN12 was found to impede the ubiquitination of PDLIM2. Further exploration based on mass spectrometry results hinted at the potential involvement of OTUD6B, functioning as a deubiquitinase, in this process. To investigate the role of circPTPN12 in this context, we conducted immunoprecipitation experiments and observed reduced binding of PDLIM2 and OTUD6B upon circPTPN12 knockdown, whereas overexpression of circPTPN12 resulted in increased binding of PDLIM2 and OTUD6B. We discovered that circPTPN12 serves as a scaffold, facilitating the interaction between PDLIM2 and OTUD6B, promoting PDLIM2 deubiquitination, maintaining its expression, and exerting its anti-cancer function.

The regulation of circRNA biogenesis is intricate. ESRP1 plays a pivotal role in the generation of various circRNAs [38–40]. Through the utilization of mass spectrometry analysis on proteins derived from RNA probes directed at the flanking intron of circPTPN12, we successfully pinpointed ESRP1 as a splicing factor intricately engaged in controlling the formation of circPTPN12. ESRP1 is acknowledged for its tumor-suppressive attributes. This reciprocal interaction between ESRP1 and circPTPN12 forms a tumor-associated regulatory pathway.

While our study delineated the inhibitory role of circPTPN12 in HCC, considering the initial screening of a limited number of HCC tissues, we acknowledge the possibility of other dysregulated circRNAs contributing to HCC proliferation or apoptosis. Thus, further exploration is essential to elucidate the broader landscape of dysregulated circRNAs in HCC.

## Conclusion

Our investigation highlights circPTPN12’s role in suppressing tumor progression within HCC by impeding cell proliferation and fostering apoptosis. Through the formation of a circPTPN12/PDLIM2/OTUD6B protein-RNA ternary complex that upholds PDLIM2 expression, circPTPN12 orchestrates modulation of the NF-κB signaling pathway, consequently facilitating the PDLIM2-mediated ubiquitination degradation of the P65 protein. Additionally, ESRP1 is identified as a regulator of circPTPN12 expression in HCC. This study significantly contributes to our comprehension of circRNAs in HCC, potentially unveiling valuable insights for diagnostic biomarkers and therapeutic targets in human HCC diagnosis and treatment.

## Materials and methods

### Tissues and cell lines

50 pairs of hepatocellular carcinoma (HCC) tissues alongside adjacent normal liver tissues were sourced from the First Affiliated Hospital at Nanjing Medical University. These tissue samples were acquired intraoperatively from patients, excluding those who had undergone anti-tumor therapy. The study received approval from our hospital’s ethics committee. The HCC cell lines employed in this study were obtained from the Cell Bank of Type Culture Collection in Shanghai, China. Primary human hepatocytes were obtained from patients undergoing hepatectomy using a modified two-step collagenase perfusion technique [41]. These cell lines were maintained in Dulbecco’s modified Eagle’s medium supplemented with 10% fetal bovine serum (Gibco, NY, USA) and cultured under standard conditions at 37 °C with 5% CO2.

### QRT-PCR

RNA extraction from both HCC tissues and cell lines, as previously described [42], was conducted using TRIzol reagent (Invitrogen, USA). Subsequently, the isolated RNA from each sample underwent reverse transcription to generate complementary DNA (cDNA). This cDNA served as a template for RT-qPCR analysis. The expression levels of the human GAPDH genes were employed as a reference to normalize the expression of both circular RNA (circRNA) and messenger RNA (mRNA). Gene expression quantification was performed utilizing the 2 − ΔΔCt method. Primer sequences utilized in this study can be found in Supplementary Table 1.

### Whole-transcriptome sequencing (RNA-seq)

RNA-seq was performed on cells transfected separately with circPTPN12 and a negative control. Library preparation involved utilizing the TruSeq Stranded Total RNA kit, and subsequent sequencing was conducted on an Illumina HiSeq™ 2500 platform. The sequencing generated paired-end reads of 150 and 125 base pairs. The RNA-seq and data analysis processes were carried out by Oebiotech (Shanghai OEbiotech Co., Ltd.) (Table S2).

### Nuclear cytoplasmic fractionation, RNase R, and actinomycin D treatment

Nuclear and cytoplasmic fractions were separated using the PARIS™ kit (Invitrogen, USA). RNaseR (2 U/µg, Epicentre, USA) was incubated with the RNAs at 37 °C for ten minutes. Subsequently, the RNAs treated with RNaseR underwent RT-qPCR analysis. Furthermore, RNA extracted from HCC cells underwent treatment with culture medium supplemented with 2 µg/ml actinomycin D (an inhibitor of DNA transcription and replication, Cell Signaling Technology, USA). Control samples treated with equivalent DMSO were also subjected to RT-qPCR analysis.

### Cycloheximide (CHX) treatment

HCC cells were cultured in the presence of cycloheximide (CHX), a protein synthesis inhibitor obtained from Selleck (China). Different treatment durations—0 h, 12 h, 18 h, and 24 h—utilizing a medium containing 25 µg/mL of CHX were administered. Proteins were subsequently extracted from the treated cells and analyzed via western blotting to assess the effects of CHX treatment on protein expression at various time points.

### RNA FISH

Custom-designed Cy3-labeled probes specific to circPTPN12 were synthesized by Sevicebio (Wuhan, China) to facilitate the precise localization of circPTPN12 in both HCC cells and tissues. The samples underwent analysis using an Olympus microscope (Tokyo, Japan). The specific probe sequence utilized in the Fluorescence. In Situ Hybridization (FISH) assay for circPTPN12 was: 5’-AGGCCATTACAATGATCTGCAATGAATACAAAT-3’.

### Transfection experiment

Human HCC MHCC-97 H and Huh7 cells were subjected to transfection using lentiviruses to induce overexpression of circPTPN12 or a scrambled control (circPTPN12/Vector) obtained from GenePharma Co., Ltd. (Shanghai, China). Additionally, specific short interfering RNA (siRNA) sequences targeting circPTPN12, ESRP1, OTUD6B, PDLIM2, and P65, along with their respective negative controls (NCs), were constructed by RiboBio Biological Technology (Guangzhou, China). The truncated PDLIM2 plasmids of PDLIM2 with a C-terminus 3× Flag tag, plasmids of P65, plasmids of ESRP1 and plasmids of OTUD6B with His tag were synthesized by Corues Bio (Nanjing, China). Lipofectamine 3000 (Invitrogen) was used as the transfection reagent. Detailed sequences utilized in this study can be found in Supplementary Table 1.

### CCK-8 assay

Cell proliferation was assessed using the CCK-8 assay kit from Dojindo (Japan). Cells were seeded at a density of 1.0 × 10^3 cells/well in a 96-well plate and cultured for specific time intervals: 24, 48, 96, 120, and 148 h. At each time point, 10 µl of the CCK-8 assay solution was added to each well and incubated for 2 h in darkness. The absorbance of each well at OD450 nm was measured using an enzyme immunoassay analyzer from Thermo Fisher Scientific, Inc. (Waltham, MA, USA) to evaluate cellular activity and proliferation.

### Clone formation assay

Stably transfected Huh7 and MHCC-97 H cells were seeded at a density of 800 cells per well in six-well plates and cultured in DMEM supplemented with 10% fetal bovine serum (FBS). After a two-week incubation period, the plates were harvested. Colonies were fixed using 4% paraformaldehyde (Sevicebio, Wuhan, China) for 25 min and subsequently stained with 0.1% crystal violet (Beyotime). Following a wash with phosphate-buffered saline (PBS), the colonies were imaged and counted to assess their growth and proliferation.

### EdU assay

HCC cells were evenly distributed in 24-well plates and cultured in DMEM. Following this, cells were treated with Edu solution obtained from Beyotime, incubated for 2 h, and processed according to the manufacturer’s instructions. The specimens were visualized and quantified using a microscope from Olympus (Tokyo, Japan) to evaluate cellular proliferation based on Edu incorporation.

### Western blot analysis

Huh7 and MHCC-97 H cells were lysed using RIPA buffer containing a protease and phosphorylation inhibitor cocktail obtained from New Cell & Molecular Biotech (Suzhou, China). Subsequently, the total protein extracts were separated by SDS-PAGE and transferred onto PVDF membranes (Millipore, USA). These membranes were then swiftly blocked using Quick Block™ Blocking Buffer for Western Blot (Beyotime, Shanghai, China) within 15 min. Following a wash with TBST, the membranes were incubated with specific primary antibodies. The next day, HRP-labeled secondary antibodies were applied, followed by visualization of the antigen-antibody complexes using an enhanced chemiluminescence detection system. Details of all antibodies utilized in this study can be found in Supplementary Table 3.

### Flow cytometry assay

A FITC Annexin V Apoptosis Detection Kit (Vazyme) was employed to determine the apoptotic cell ratio. In summary, 5 × 10^5 cells were harvested, washed twice with PBS, and suspended in 300 µL of binding buffer. Subsequently, the cells were stained with 3 µL of Annexin V-phycoerythrin and 3 µL of propidium iodide. Following a 15-minute incubation period, flow cytometry analysis was performed using a FACSCalibur™ Flow Cytometer (BD Biosciences, SanJose, CA, USA) to assess the stained cells.

### Biotin-labelled RNA pull-down and mass spectrometry

Biotinylated circPTPN12 probes, designed by RiboBio (Guangzhou, China), were used in this study. Initially, 1 × 10^7 cells underwent lysis. Subsequently, 50 µL of beads were incubated with an equal volume of circPTPN12 probe for 2 h. Following two washes with 50 µL of Tris, the Huh7 cell lysates, previously treated with probe-coated beads, were incubated overnight at 4 °C. RNA-protein complexes were then eluted and extracted for subsequent analysis via western blotting and mass spectrometry. Silver staining was performed following the manufacturer’s instructions using the Fast Silver Stain Kit from Beyotime. The protein bands specific to the circPTPN12 probe group, in contrast to the control group, were analyzed using the Q Exactive mass spectrometer (Thermo Fisher Scientific, CA, USA). The results are presented in Supplementary Tables 4 and Supplementary Table 5.

### RIP assay

The RNA immunoprecipitation assay was performed using the Magna RIP RNA-Binding Protein Immunoprecipitation Kit from Millipore (USA), following the manufacturer’s instructions. Purified RNA obtained from cellular samples was subsequently utilized for qRT-PCR analysis.

### Animal experiments

Four-week-old male nude mice were procured from the Animal Core Facility of Nanjing Medical University (Nanjing, China) for in vivo tumor growth experiments. These mice were randomly assigned into four groups, with five mice in each group.

Initially, 2 × 10^6 Huh7 cells suspended in 100uL PBS were subcutaneously injected into the dorsal flank of each mouse. After two weeks, mice with observable tumors were randomly divided into two groups. One group received intratumoral injections of 50 nmol of cholesterol-conjugated si-NC or si-circPTPN12 three times per week for a duration of 2 weeks. The remaining mice were injected with MHCC-97 H cells that stably overexpressed circPTPN12 or a vector control.Tumor volumes and weights were monitored at various time points throughout the study. Upon completion, all samples were fixed in 4% paraformaldehyde and subjected to staining using immunohistochemistry (IHC) techniques.

### IHC

The xenografts tissues underwent a process of dewaxing and rehydration using an ethanol gradient for preparation. Subsequently, they were subjected to heat-induced antigen retrieval in citrate buffer (10 mM, pH 6.0) at 95 °C for 20 min. Following this, the samples were incubated overnight at 4 °C with polyclonal antibodies targeting P65 (Proteintech), Ki67 (Cell Signaling Technology), and PDLIM2 (Novus Biologicals), followed by incubation with secondary antibodies for 1 h. Evaluation of staining involved assessing the proportion of positively stained cells. The intensity of staining for each sample was graded on a scale ranging from 0 (negative) to 3 (strong), reflecting the absence or presence of weak, moderate, or strong staining, respectively.

### Immunofluorescence staining

HCC cells, fixed with 4% paraformaldehyde (PFA) for 20 min, underwent permeabilization using 1% Triton X-100 for 30 min. Following a 30-minute block with 10% donkey serum, primary antibodies were applied and incubated for 2 h at 37 °C. After PBS washing, fluorescence-conjugated secondary antibodies were added and incubated at 37 °C for 1 h. Sealing was performed, and observation was conducted using Olympus microscope (Tokyo, Japan).

### Statistical analysis

In this study, numerical data and histograms are shown as the mean ± SEM. Specific statistical parameters for the analyses are provided in the figure legends. Each experiment was conducted a minimum of three times. Data analysis was conducted utilizing Prism 8 software (GraphPad Software, La Jolla, CA, USA). Student’s t-test was employed to assess differences between means. The correlation between circPTPN12 and ESRP1 expression levels was determined using Pearson’s test (r, P). For analyzing the relationship between circPTPN12 expression and clinicopathological parameters, the P value was determined using the Pearson chi-square test. For survival analysis, the overall survival (OS) rate was calculated employing the Kaplan-Meier method and compared using the log-rank test. Statistical significance was denoted by *p < 0.05, **p < 0.01, and ***p < 0.001. These levels were considered indicative of significant differences in the observed data.

### Electronic supplementary material

Below is the link to the electronic supplementary material.

Supplementary Material 1

Supplementary Material 2

Supplementary Material 3

Supplementary Material 4

Supplementary Material 5

Supplementary Material 6

Supplementary Material 7

Supplementary Material 8

Supplementary Material 9

Supplementary Material 10

Supplementary Material 11

Supplementary Material 12

Supplementary Material 13

## Data Availability

All data generated or analyzed during this study are included either in this article or in the supplementary information files.

## Abbreviations

HCC: Hepatocellular carcinoma
circRNA: Circular RNA
OS: Overall survival
FISH: Fluorescence in situ hybridization
cDNA: Complementary DNA
gDNA: Genomic DNA
qRT-PCR: Quantitative real-time PCR
siRNAs: Short interfering RNAs
ceRNA: Competitive endogenous RNA
RBP: RNA binding protein
RIP: RNA immunoprecipitation
DMEM: Dulbecco’s Modified Eagle Medium
FBS: Fetal bovine serum
χ2 test: Chi-square test
DAPI: 4′, 6-diamidino-2-phenylindole
PBS: phosphate-buffered saline
bp: Base pair
PDLIM2: PDZ-LIM domain-containing protein 2
OTUD6B: Ovarian tumor domain-containing 6B
MS: Mass spectrometry
IHC: Immunohistochemistry
ESRP1: Epithelial splicing regulatory protein 1
