# Supplementary material for: ESRP1-mediated biogenesis of circPTPN12 inhibits hepatocellular carcinoma progression by PDLIM2/ NF-κB pathway
Source: Mol Cancer. 2024 Jul 11;23:143. doi: 10.1186/s12943-024-02056-1 (PMC11238376; doi:10.1186/s12943-024-02056-1)
Supplement: Supplementary file 1 — Supplementary Material 1 [file 12943_2024_2056_MOESM1_ESM.docx]

| hsa-circ-0003764 | F:5’- ATTTGTATTCATTGCAGATCAT-3’ |
| --- | --- |
|  | R:5’-GCTTGTTCATCCTCACAAGA-3’ |
| hsa-circ-0002458 | F:5’- CACCATTTAAAATTTCTTGTTT-3’ |
|  | R:5’- TGCTAAAGGTCCTTGAGTTGC-3’ |
| hsa-circ-0005197 | F:5’-CTTACTTGAATTTCAAAATTT-3’ |
|  | R:5’-TGCTAAAGGTCCTTGAGTTGC-3’ |
| hsa-circ-0007411 | F:5’-GCCAGACCATGATGTTCCTTC-3’ |
|  | R:5’-ATTTAACTCGGCTGTGATCAACT-3’ |
| hsa-circ-0008139 | F:5’-GCAACTCAAGGACCTTTAGCA-3’ |
|  | R:5’-TAACTCGGCTGTGATCAACACA-3’ |
| hsa-circ-0008901 | F:5’-CATTTAAAATTTCTTGTCGGT-3’ |
|  | R:5’-TGGCTGTGGGATATATCTTTT-3’ |
| PTPN12 | F:5’-CATATGGTGGGATACC-3’ |
|  | R:5’-GCTTCACTCGAGAAGCAGCT-3’ |
| ESRP1 | F:5’-ACGGAGGACTGCAAAGAAGA-3’ |
|  | R:5’-CTGACATGAAGCTGCCCATC-3’ |
| P65 | F:5’CGCGGATCCGCCACCATGGACGAACTG-3’ |
|  | R:5’-CCGCTCGAGTTAGGAGCTGATCTG-3’; |
| PDLIM2 | F:5’- AGAGGAGTTTATATATATTTAGG -3’; |
|  | R:5’-TACCTAACAACCCTCTCTCC-3’; |
| OTUD6B | F:5’-TGAGGGGTTTTGGATTAGATG-3’ |
|  | R:5’-AATGGCAGAAAGTCTTCCACA-3’ |
| GAPDH | F: 5’-GTGAAGCAGGCGTCGGA-3’ |
|  | R: 5’-AGCCCCAGCGTCAAAGG-3’ |
| GAPDH (divergent) | F: 5’-TGTACCATCAATAAAGTACCCTGTG-3’ |
|  | R: 5’-AAATCCGTTGACTCCGACCT-3’ |
| U6 | F: 5’-CTCGCTTCGGCAGCACA-3’ |
|  | R: 5’-AACGCTTCACGAATTTGCGT-3’ |

**Table. S1.** The sequences of primer, siRNA and RNA pulldown probes

Primers

SiRNA

| hsa-circ-0003764: | Sense: 5’-GTATTCATTGCAGATCATTGT-3’ |
| --- | --- |
|  | Anti-sense:5’-TTCGGAGCCTATCGAGCCAC-3’ |
| ESRP1 | Sense:5’- GCAGCTTCATGTCAGGCAATT  -3’ |
|  | Anti-sense:5’-TTCTCCGAACGTGTCACGTTT-3’ |
| P65 | Sense: 5’- GGCGAGAGGAGCACAGATACC-3’ |
|  | Anti-sense: 5’- TATCTGTGCTCCTCTCGCCTG-3’ |
| PDLIM2 | Sense：5′- GGACAGCTCCTTGGAAGTG -3′, |
|  | Anti-sense： 5′-TACGATAGTTCGATCGTTT-3′ |
| OTUD6B | Sense：5′- GTTTTGGATTAGATGATATCTAAGG-3′ |
|  | Anti-sense 5′-GGAAATCTTTCTGCGTGCTGTTTCC-3′ |

RNA pulldown probes

| hsa-circ-0003764: | Sense:5’-GCCATTACAATGATCTGCAA-3’ |
| --- | --- |
|  | Anti-sense:5’-ATCAGCATTATCCGACTGAA-3’ |
| PTPN12 Intron 4 | Sense:5’- AATCTTTCAAAGACCAGAGC -3’ |
|  | Anti-sense: 5’- AGATCATAACCGCTAGACT-3’ |
| PTPN12 Intron 8 | Sense:5’- ATGCAGATAACAAGTCCCTT-3’ |
|  | Anti-sense:5’- AGATCATAACCGCTAGACT-3’ |
