# Supplementary material for: ESRP1-mediated biogenesis of circPTPN12 inhibits hepatocellular carcinoma progression by PDLIM2/ NF-κB pathway
Source: Mol Cancer. 2024 Jul 11;23:143. doi: 10.1186/s12943-024-02056-1 (PMC11238376; doi:10.1186/s12943-024-02056-1)
Supplement: Supplementary file 3 — Supplementary Material 3 [file 12943_2024_2056_MOESM3_ESM.docx]

| **REAGENT or RESOTRCE** | **SOTRCE** | **IDENTIFIER** |
| --- | --- | --- |
| **Antibodies** | | |
| Rabbit anti-p65 | Proteintech | 10745-1-AP |
| Rabbit anti-PDLIM2 | Novus Biologicals | NBP1-85800 |
| Rabbit anti-ESRP1 | Proteintech | 21045-1-AP |
| Rabbit anti-OTUD6B | Proteintech | 25430-1-AP |
| Rabbit anti-Bcl2 | CST | 4223T |
| Rabbit anti-Bax | CST | 2772T |
| Rabbit anti-Flag | CST | 14793T |
| Rabbit anti-His | CST | 12698T |
| Rabbit anti-Ki67 | CST | 9129T |
| Rabbit Anti- GADPH | CST | 5174T |
| Rabbit anti- IKK beta | abcam | ab124957 |
| Rabbit Anti-IKK alpha + IKK beta | abcam | ab194528 |
| Rabbit Anti-IKK alpha | abcam | ab32041 |
| Rabbit Anti-IkBa | ABclonal | A19714 |
| Rabbit Anti-Phospho-IκBα | ABclonal | AP0707 |
| Rabbit anti-ubiquitin | Proteintech | 10201-2-AP |
| Anti-rabbit IgG, HRP-linked Antibody | CST | 7074S |
| Alexa Fluor 594-conjugated goat anti-Rabbit IgG | Invitrogen | A-11012 |
| Alexa Fluor 488-conjugated goat anti-Rabbit IgG | Invitrogen | A-11008 |
| Anti-IgG | Abcam | ab172730 |

**Table. S3. Antibodies**
