# Supplementary figures and images for: ESRP1-mediated biogenesis of circPTPN12 inhibits hepatocellular carcinoma progression by PDLIM2/ NF-κB pathway
Source: Mol Cancer. 2024 Jul 11;23:143. doi: 10.1186/s12943-024-02056-1 (PMC11238376; doi:10.1186/s12943-024-02056-1)

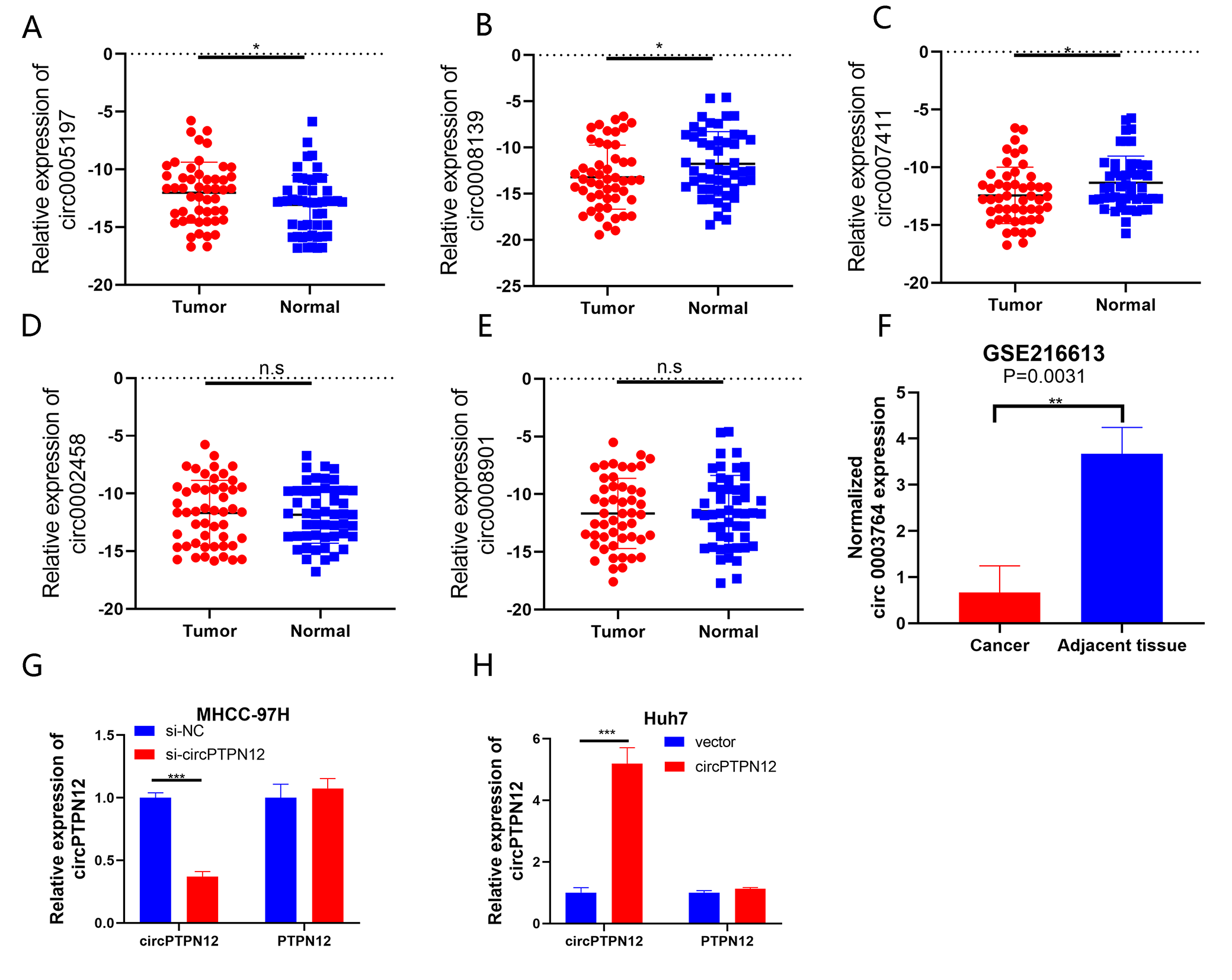

Supplement: Supplementary file 8 — Supplementary Material 8 [file 12943_2024_2056_MOESM8_ESM.tif]

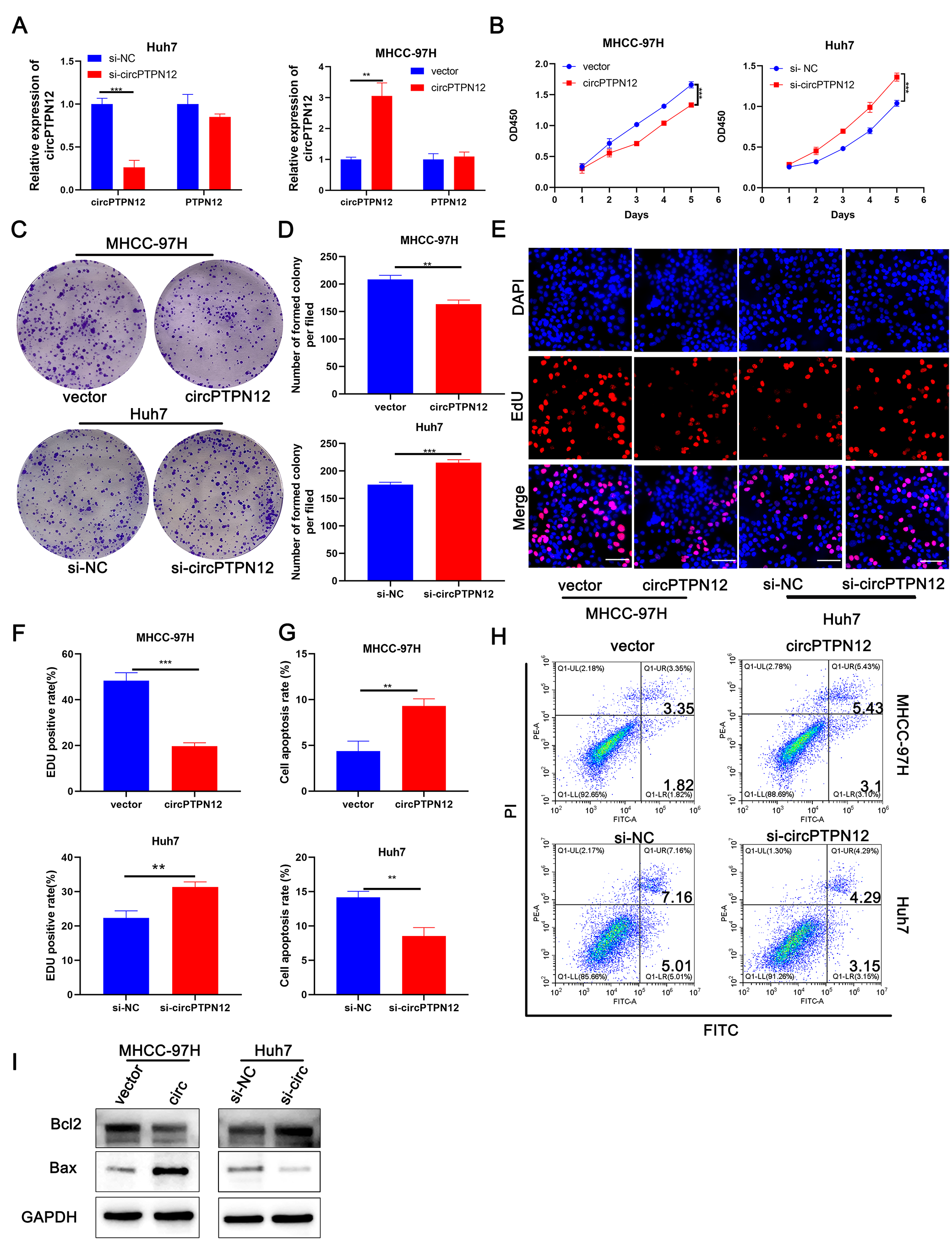

Supplement: Supplementary file 9 — Supplementary Material 9 [file 12943_2024_2056_MOESM9_ESM.tif]

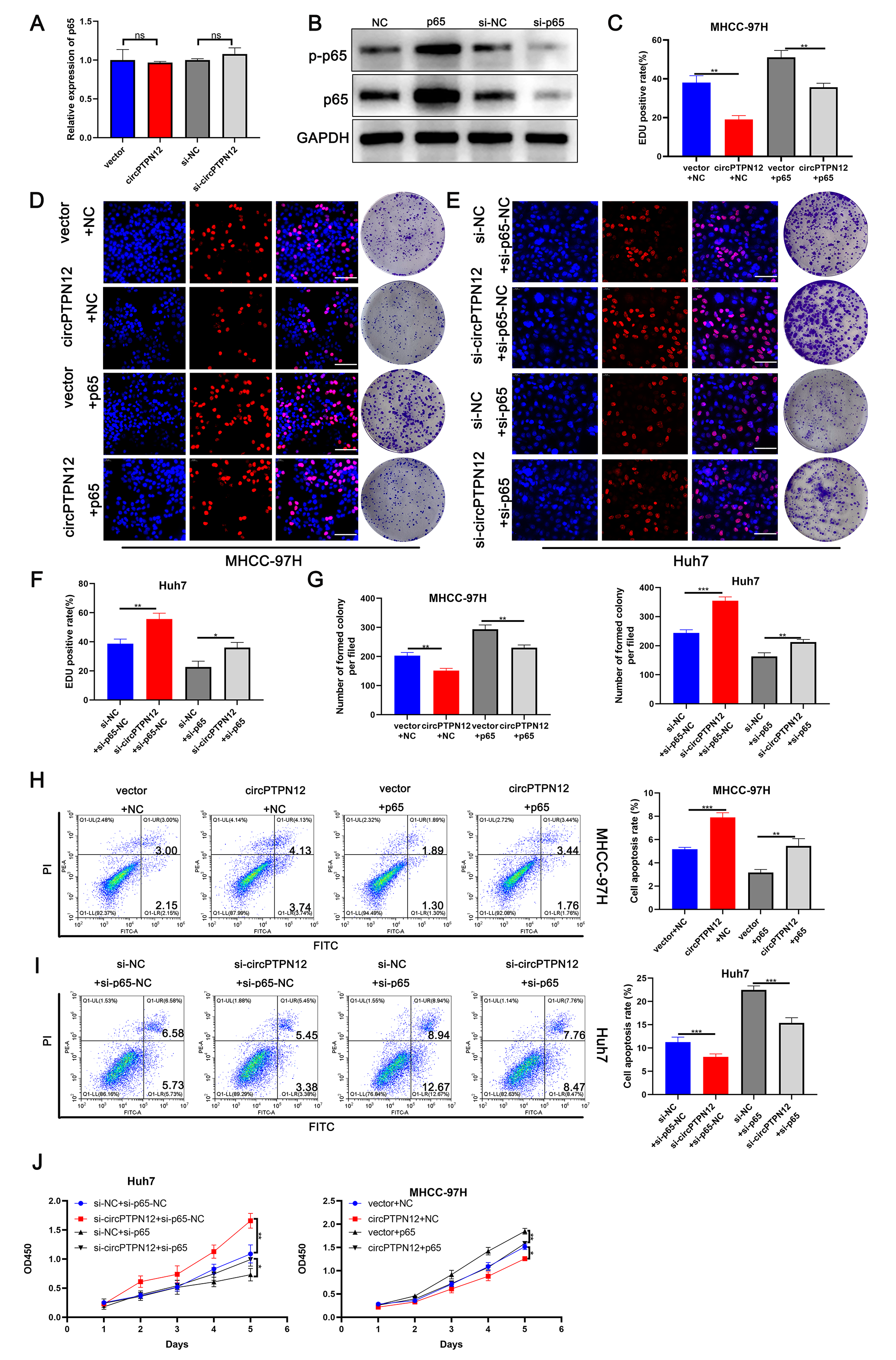

Supplement: Supplementary file 10 — Supplementary Material 10 [file 12943_2024_2056_MOESM10_ESM.tif]

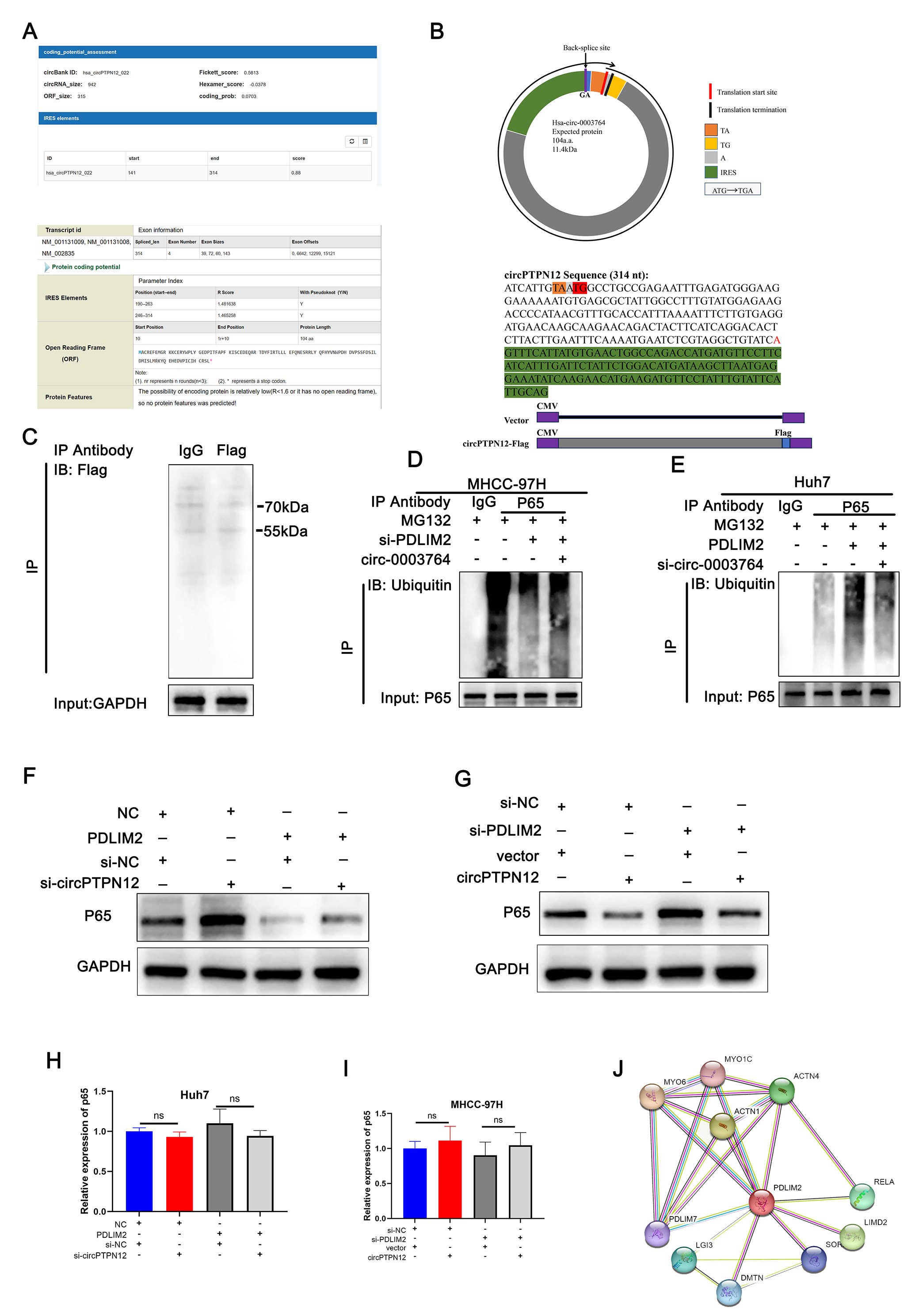

Supplement: Supplementary file 11 — Supplementary Material 11 [file 12943_2024_2056_MOESM11_ESM.tif]

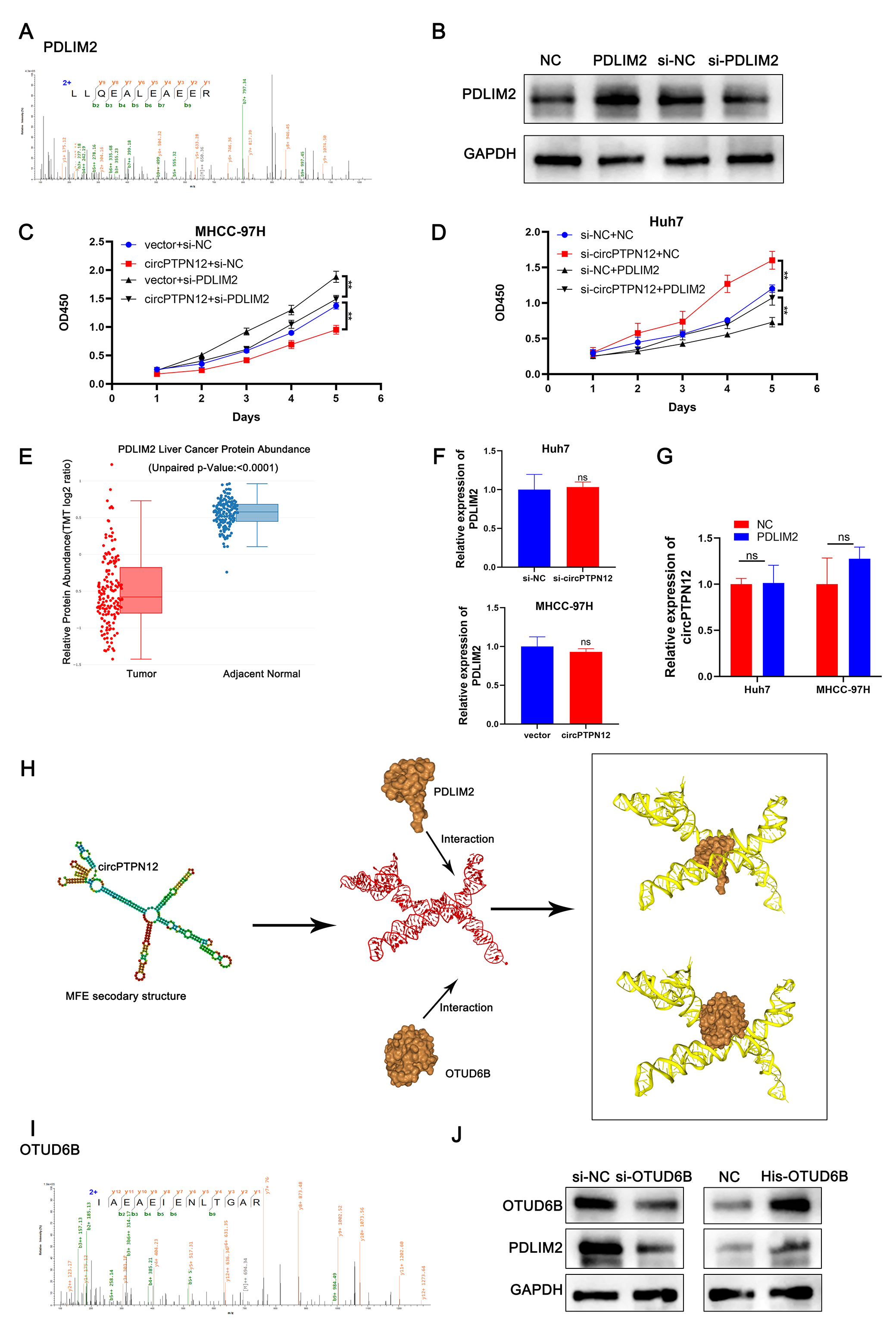

Supplement: Supplementary file 12 — Supplementary Material 12 [file 12943_2024_2056_MOESM12_ESM.tif]

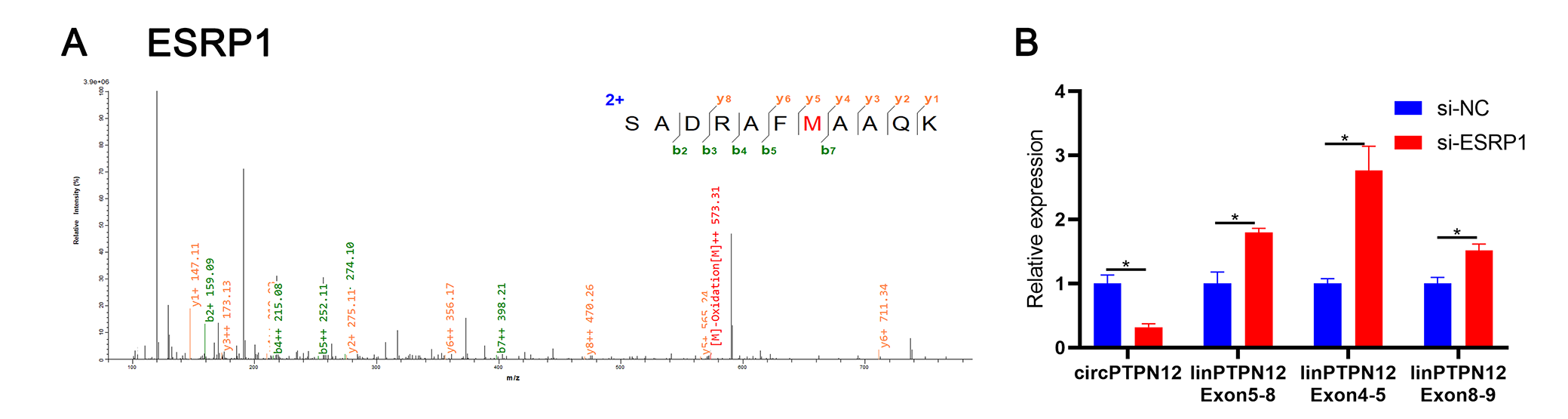

Supplement: Supplementary file 13 — Supplementary Material 13 [file 12943_2024_2056_MOESM13_ESM.tif]
